# Deciphering the electrochemical sensing capability of novel Ga_12_As_12_ nanocluster towards chemical warfare phosgene gas: insights from DFT†

**DOI:** 10.1039/d3ra05086f

**Published:** 2023-10-02

**Authors:** Muhammad Javed, Muhammad Usman Khan, Riaz Hussain, Sarfraz Ahmed, Tansir Ahamad

**Affiliations:** a Department of Chemistry, University of Okara Okara-56300 Pakistan usman.chemistry@gmail.com usmankhan@uo.edu.pk riazhussain@uo.edu.pk; b Wellman Center for Photomedicine, Harvard Medical School, Massachusetts General Hospital Boston MA 02114 USA; c Department of Chemistry, College of Science, King Saud University Saudi Arabia

## Abstract

The applications of 3D inorganic nanomaterials in environmental and agriculture monitoring have been exploited continuously; however, the utilization of semiconductor nanoclusters, especially for detecting warfare agents, has not been fully investigated yet. To fill this gap, the molecular modelling of novel inorganic semiconductor nanocluster Ga_12_As_12_ as a sensor for phosgene gas (highly toxic for living things and the environment) is accomplished employing benchmark DFT and TD-DFT investigations. Computational tools have been applied to explore different adsorption sites and the potential sensing capability of the Ga_12_As_12_ nanoclusters. The calculated adsorption energy (−21.34 ± 2.7 kcal mol^−1^) for ten selected complexes, namely, Pgn–Cl@4m-ring (MS1), Pgn–Cl@6m-ring (MS2), Pgn–Cl@XY66 (MS3), Pgn–O@4m-ring (MS4), Pgn–O@XY66 (MS5), Pgn–O@XY64 (MS6), Pgn–O@Y (MS7), Pgn–planar@Y (MS8), Pgn–planar@X (MS9), and Pgn–planar@4m-ring (MS10), manifest the remarkable and excessive adsorption response of the studied nanoclusters. The explored molecular electronic properties, such as interaction distance (3.05 ± 0.5 Å), energy gap (∼2.17 eV), softness (∼0.46 eV), hardness (1.10 ± 0.01 eV), electrophilicity index (10.27 ± 0.45 eV), electrical conductivity (∼1.98 × 10^9^), and recovery time (∼3 × 10^−12^ s^−1^) values, ascertain the elevated reactivity and an imperishable sensitivity of the Ga_12_As_12_ nanocluster, particularly for its complex MS8. QTAIM analysis exhibits the presence of a strong electrostatic bond (positive *∇*^2^*ρ*(*r*) values), electron delocalization (ELF < 0.5), and a strong chemical bond (because of high all-electron density values). In addition, NBO analysis explores the lone pair electron delocalization of phosgene to the nanocluster stabilized by intermolecular charge transfer (ICT) and different kinds of non-covalent interactions. Also, the green region existence expressed by NCI analysis (between the nanocluster and adsorbate) stipulate the energetic and dominant interactions. Furthermore, the UV-Vis, thermodynamic analysis, and density of state (DOS) demonstrate the maximum absorbance (562.11 nm) and least excitation energy (2.21 eV) by the complex MS8, the spontaneity of the interaction process, and the significant changes in HOMO and LUMO energies, respectively. Thus, the Ga_12_As_12_ nanocluster has proven to be a promising influential sensing material to monitor phosgene gas in the real world, and this study will emphasize the informative knowledge for experimental researchers to use Ga_12_As_12_ as a sensor for the warfare agent (phosgene).

## Abstract 2

The electrochemical sensing capability of novel Ga_12_As_12_ nanocluster towards chemical warfare phosgene gas (highly toxic for living things and the environment) is successfully explored employing DFT and TD-DFT computations.

## Graphical abstract

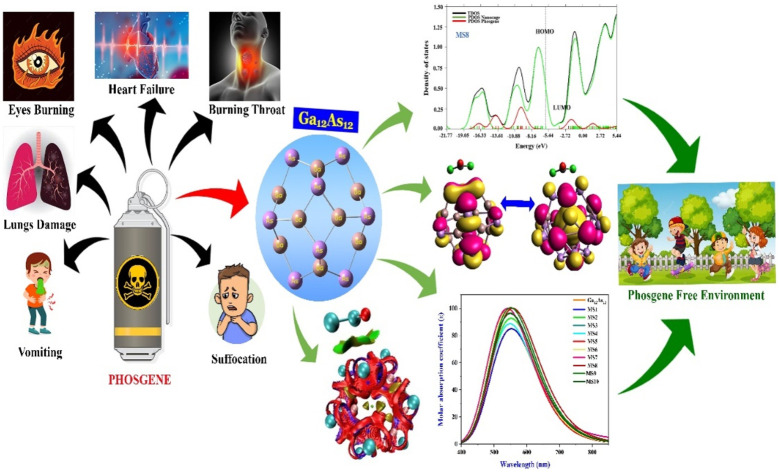

## Introduction

Warfare agents are a serious threat towards populations, mainly in urban areas. They are more toxic, reactive, and mostly produced in industries. Phosgene gas, having the molecular formula COCl_2_, is one of the most poisonous warfare agents and was used in World War I as a chemical weapon.^1^ It can be used to produce polyurethanes, isocyanate, plastics, dyes, pesticides, and pharmaceutical products. It is also used in the industry as a protective gas to produce dimethyl diphenyl urea. The annual production of phosgene is about 2 million tons, which is used to produce fine chemicals and polymers. It is very dangerous to store, transport, and utilize phosgene gas due to its high volatility and toxicity.^2,3^ Phosgene gas attained a reputation for the first time when its exposure caused about 80% of deaths due to all warfare agents during World War I. It can cause irritation in the mucosal membrane and lung damage when its concentration is below 3 ppm, but at high concentration above 150 ppm, it can cause latent non-cardiogenic pulmonary edema, and it is also life-threatening.^4,5^

In the recent 20 years, after the discovery of carbon-based nanoparticles and nanoclusters, several efforts have been made to discover other nanoparticles that include metallic nanoparticles, ceramic-type nanoparticles, and semiconductor nanoparticles.^6^ These nanoparticles have gained more attention from researchers to detect, absorb, and destroy more toxic chemicals and warfare agents because of their unique properties.^7^ The porous surfaces, the large surface-to-volume ratio, and the optical, electrical, mechanical, and magnetic properties of these nanoparticles make them unique. The particles also have a large HOMO–LUMO gap and special chemical and physical properties.^8^ Therefore, they have a wide range of applications in the chemical industry, medicine, electronics, and aviation. The most important application of these nanoparticles and nanoclusters is because of their sensing property to detect and absorb poisonous and toxic gases and warfare agents.^9^ The sensing property, reactivity, physical and chemical properties, and molecular structure of nanoparticles and nanoclusters are examined by density functional theory (DFT).^10–13^

Literature review has shown the adsorption of toxic gases, especially warfare agents, by many fullerene-like inorganic nanoclusters due to their large HOMO–LUMO gap.^14,15^ The adsorption, sensing, and detection of phosgene gas by different nanomaterials have been investigated. For instance, Ti, Ni, and Cu-decorated borospherene nanocluster,^16^ Al_12_N_12_ nanocluster as a potential sensor,^1^ different angle oriented boron nitride (BN) nanocones with 60°, 120°, 180°, and 240° disclination angles,^17^ Sc-doped BN nanoclusters,^18^ pristine and Cu-decorated B_12_N_12_ nanocluster,^19^ and Ca_12_O_12_, Mg_12_O_12_, and Al_12_N_12_ nanomaterials^20^ have been investigated to be good sensing nanomaterials for phosgene gas.

Several inorganic nanoclusters such as B_12_P_12_,^21^ Ga_12_As_12_,^22,23^ and Al_12_P_12_ (ref. 24) have been investigated to explore their potential applications in non-linear optics,^25,26^ the photoelectrochemical solar energy conversion,^27,28^ and in sensing devices for the detection of wide range of chemicals.^29,30^ These fullerene-like nanoclusters have been found to show intermolecular interactions with diazomethane^31^ and for the adsorption of alkali and alkaline earth metals.^32^ They also exhibited interaction with halo-methane^33^ and used it for the adsorption of toxic gasses.^19^ They have been employed for 4-aminopyridine drug delivery and the adsorption of the sorbic acid drug using density functional theory.^34,35^

According to the best of our literature investigation and knowledge, no specific investigations has been fully done regarding the sensing capability of the Ga_12_As_12_ nanocluster for toxic chemical warfare agents because the detection of dangerous chemical warfare agents by a fast and accurate method is the need of current situation, which urged us to investigate the potential sensing of the Ga_12_As_12_ nanocluster toward phosgene gas. For this purpose, the sensing and adsorption of phosgene gas with different orientations on different adsorption sites of Ga_12_As_12_ nanocluster have been studied comprehensively by applying benchmark DFT and TD-DFT computations.

Various analyses such as HOMO–LUMO, natural bond orbitals (NBO), quantum theory of atoms in a molecule (QTAIM), non-covalent interactions (NCI), molecular electrostatic potential (MEP), density of state (DOS), and thermodynamic analysis have been conducted to investigate the intermolecular interactions and charge distributions between the Ga_12_As_12_ nanocluster and phosgene gas and to elucidate the sensing capability of the Ga_12_As_12_ nanocluster. With the evidence of the current investigations, it is optimistically proposed that the Ga_12_As_12_ nanocluster will be utilized as an excellent and potential sensor for phosgene gas detection.

## Computational studies

In the current research, the geometry optimization, frequency analysis, interaction energy, HOMO–LUMO energy gap, and Fermi level energy calculations of the Ga_12_As_12_ nanocluster and its ten selected complexes (MS1–MS10) were performed using DFT and TD-DFT at the B3LYP-D3/6-31G(d,p) level of theory using GaussView and Gaussian 09 suite of programs.^36,37^ This B3LYP-D3/6-31G(d,p) level of theory was selected by comparing the bond length of the optimized structure of the nanocluster by different functionals with the already reported data.^32^ Utilizing the same functional, the NBO^38^ and MEP analyses were performed to analyze the charge distributions of the nanocluster complexes and the net charge on phosgene gas. The DOS analysis of the nanocluster and complexes was performed using the Multiwfn 3.8 program.^39^ To find out the electronic transition within the nanocluster and complexes, HOMO–LUMO plots have been obtained using the Avogadro-1.2.0 program.^40^ The topological analysis quantum theory of atoms in molecules (QTAIM) and non-covalent interactions (NCI) analysis was used to attain more insights into the nature of the inter-atomic interactions visual molecular dynamic (VMD) 1.9.4 program.^41^ The computations of QTAIM were done *via* Multiwfn 3.8 program.^39^ A mathematical method named thermodynamic analysis (TDA) was applied to study spontaneous and non-spontaneous behavior of the system based on the interaction of work and heat with chemical reactions and variations in the physical state through the law of thermodynamics.^42,43^

## Results and discussion

### Selection of the method

The Ga_12_As_12_ nanocluster was optimized by applying three reported and widely used functionals of DFT, namely, CAM-B3LYP,^44^ B3LYP-D3,^45^ and ωB97XD,^46^ assigned with the 6-31G(d,p) basis set. The functional CAM-B3LYP demonstrates long-range interactions but does not provide dispersion correction and charge transfer excitations to a fine extent. To obtain long-range interactions and form dispersion correction, the ωB97XD functional was developed by Head-Gordon *et al.*^46^ The bond lengths of all the different sides of the nanocluster have been calculated and compared with the already reported data, as shown in Table 1.

**Table 1:** The calculated bond lengths (Å) of the Ga_12_As_12_ nanocluster at three different functionals comparison with the already reported data

Module	ML-2.420^a^	MR2.423^a^	T-2.417^a^	TL-2.484^b^	TR-2.480^b^	RB-2.426^b^	RC-2.484^b^
B3LYP-D3	2.33711	2.33711	2.33711	2.42042	2.42042	2.33711	2.42042
CAMB3LYP	2.32197	2.32197	2.32197	2.39467	2.39467	2.32197	2.39467
ωB97XD	2.31803	2.31803	2.31803	2.39027	2.39027	2.31803	2.39027

a XY64 sides of the nanocluster.

b XY66 sides of the nanocluster, ML = mid left, MR = mid right, T = top, TL = top left, TR = top right, RB = rim B, RC = rim C.

**Fig. 1:**
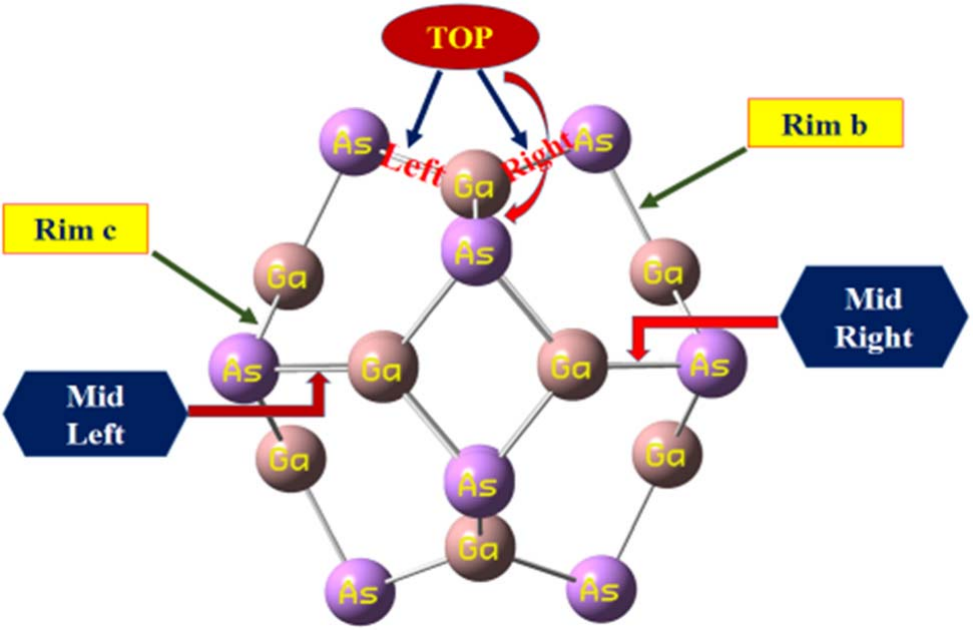
The proposed names of all the sites of the nanocluster to compare the bond lengths with the reported data.

The calculated bond lengths of the nanocluster optimized using DFT with the functional B3LYP-D3 known as D3 and GD3 with the 6-31G(d,p) basis set were found to be consistent with previously published studies.^31^ Thus, this functional developed by Grimme *et al.* was selected as the most suitable method to investigate the sensing capability of the studied system. In order to compare the bond lengths of the nanocluster structure optimized by three different functionals with the reported data, the names of all the sites of the nanocluster were proposed and are represented in Fig. 1.

### Geometry optimization

**Fig. 2:**
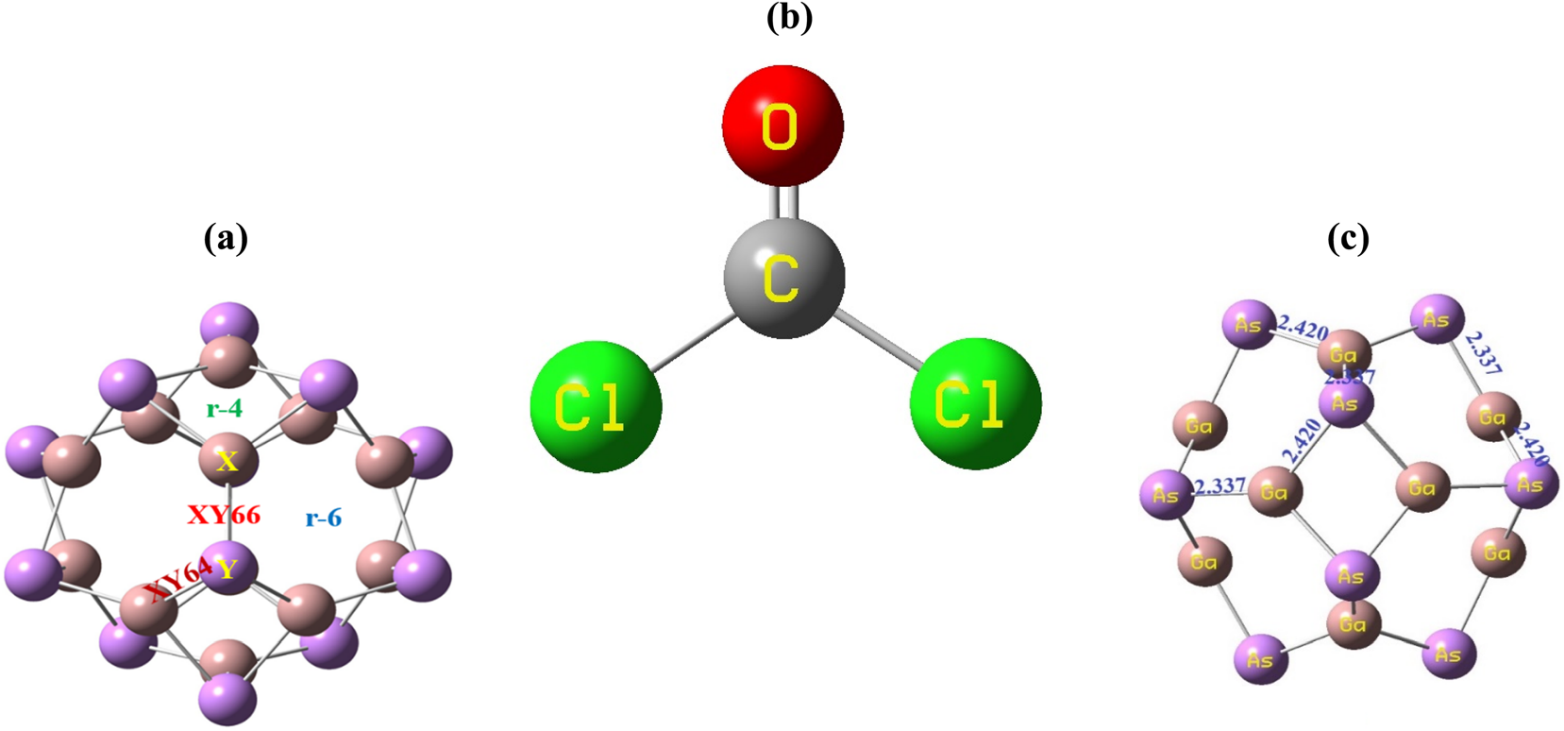
The adsorption sites of the nanocluster (a), the optimized structure of the gas (b) and the nanocluster (c).

The geometry optimization of the Ga_12_As_12_ nanocluster, phosgene gas, and their possible complexes were carried out using DFT at the latest functional B3LYP-D3/6-31G(d,p) level of theory. The Ga_12_As_12_ nanocluster contains eight symmetric 6-membered rings and six symmetric 4-membered rings and has equal adsorption sites to sense any toxic and dangerous chemicals on their outer surface. The adsorption and sensing sites are classified as follows: on top of the X-atom, on top of the Y-atom, on top of the r-6 position, on top of the r-4 position, on top of the XY66 bond, and on top of the XY64 bond (Fig. 2a). After the optimization of the isolated nanocluster and phosgene gas, the gas is placed on the surface of the nanocluster on six different sites, as mentioned above, with three different orientations, namely, planar, oxygen toward the nanocluster, and chlorine toward the nanocluster. The gas is placed at a vertical distance between 4.7 and 5.0 Å on the outer surface of the nanocluster.

**Fig. 3:**
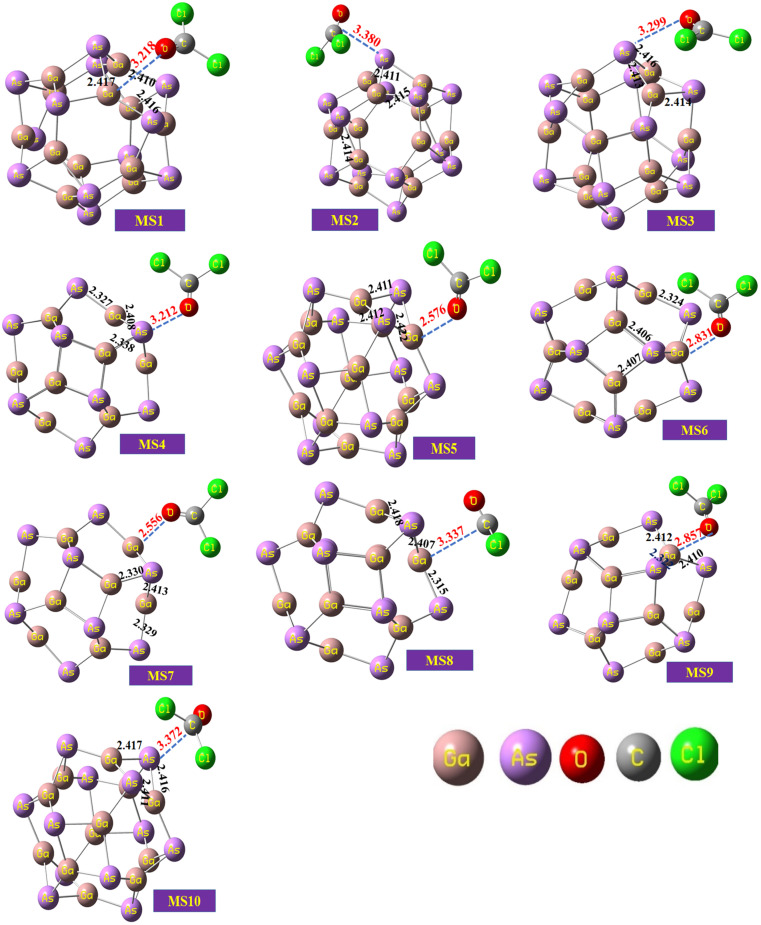
The optimized structures of the complexes of the studied system calculated at the B3LYP-D3/6-31G(d,p) level of theory.

The optimized geometry of the phosgene gas and bond lengths of the nanocluster were calculated from the optimized structures of the isolated Ga_12_As_12_ nanocluster obtained by the B3LYP-D3 and shown in Fig. 2b and c, respectively. By evaluating all possible doping sites of Ga_12_As_12_, a comprehensive investigation of the phosgene atoms interaction with Ga_12_As_12_ was carried out. Ten designed complexes with phosgene (Pgn) gas named as Pgn–Cl@4m-ring (MS1), Pgn–Cl@6m-ring (MS2), Pgn–Cl@XY66 (MS3), Pgn–O@4m-ring (MS4), Pgn–O@XY66 (MS5), Pgn–O@XY64 (MS6), Pgn–O@Y (MS7), Pgn–planar@Y (MS8), Pgn–planar@X (MS9), and Pgn–planar@4m-ring (MS10) were optimized to the true minima, as evidenced by all real frequencies. The final B3LYP-D3 optimized structures of nanocluster complexes, the interaction distances with the phosgene gas, and their bond lengths variations have been calculated and shown in Fig. 3. The interaction distance along with the interacting atoms between the nanocluster and the phosgene gas is calculated and expressed in Table S1 (ESI†). The complex having less interaction distance represents a relatively stronger interaction between the gas and the nanocluster and expresses an excellent sensing response and *vice versa*.

It has been observed that complexes MS5 and MS7 indicate the minimum interaction distances to express comparatively stronger interactions and reactivity while complexes MS2 and MS10 indicate maximum interaction distances between the phosgene gas and the surface of the Ga_12_As_12_ nanocluster. The bond distances of the nanocluster after the interaction of the gas are mentioned in Fig. 3 that can be compared with Fig. 2c to observe a variation as a result of interaction.

### Adsorption studies

The interaction energy of the nanocluster complexes with phosgene gas has been calculated to improve its reliability and find out all possible interactions. The interaction energy of the gas with nanocluster was calculated by eqn (1)–(3).^47,48^

1 *E*_ads_ = *E*_complex_ − *E*_nanocage_ − *E*_gas_

2 *E*_INT_ = *E*_complex_ − *E*_nanocage_ − *E*_gas_ + *E*_BSSE_

where *E*_ads_ is the adsorption energy of the nanocluster for the gas, *E*_INT_ is the counterpoise corrected interaction energy, *E*_complex_ is the total energy of the gas/nanocluster cluster, and *E*_nanocage_ and *E*_gas_ are the energy values of isolated nanocluster and isolated gas, respectively. *E*_BSSE_ defines the basis set superposition error of the respective complex obtained by the counterpoise approach that Bernardi and Boys established.

3 Δ*E*_BSSE_ = Δ*E*_system_ − Δ*E*_system–complex_ − Δ*E*_system–gas_

According to the above equation, the negative values of *E*_ads_ represent the stability of the formed complex and the positive values of *E*_ads_ represent the barrier in gas adsorption to the nanocluster. The interaction energy has been calculated by placing gas on the mentioned positions of the nanocluster. A minor difference in the interaction energy has been observed for the six possible positions for adsorption. Using gas with different orientations, such as planar, oxygen toward the nanocluster and chlorine toward the nanocluster, many identical results for *E*_ads_ are obtained.^49^ One result of similar values is chosen to show interaction energies for corresponding adsorption sites with three different orientations of phosgene gas. Ten complexes have been selected out of eighteen due to having identical results of adsorption energy values; these results are shown in Table 2.

**Table 2:** The adsorption energy (*E*_ads_) and counterpoise corrected interaction energy (*E*_INT_) values of the complexes and basis set superposition error (*E*_BSSE_) calculated at the B3LYP-D3/6-31G(d,p) level of theory^a^

Name	*E*_ads_	*E*_BSSE_	*E*_INT_
MS1	−24.04078	0.03223	−3.81549
MS2	−22.29381	0.02839	−4.47724
MS3	−20.27640	0.02722	−3.19817
MS4	−18.64804	0.02473	−3.13091
MS5	−20.52678	0.02545	−4.55651
MS6	−21.27789	0.02649	−4.65782
MS7	−20.62906	0.02556	−4.59190
MS8	−21.82508	0.03014	−2.56082
MS9	−21.47493	0.03129	−2.19224
MS10	−20.54560	0.02753	−3.26912

a *E*_INT_ and *E*_ads_ are in kcal mol^−1^ and BSSE in hartree.

The selection of the site for adsorption has a minor effect on other sites of corresponding energy values as in the present work; the MS1 complex shows the highest adsorption response and better sensing capability toward phosgene gas while complex MS6 represents the highest counterpoise corrected interaction energy (*E*_INT_) among all the complexes of the nanocluster. The adsorption position showing high interaction energy values indicates better sensing capability of the nanocluster's complex. Among all the adsorption sites with three different orientations, the maximum difference in their adsorption energies for all the complexes is about 5.392 kcal mol^−1^.

### Frontier molecular orbitals analysis

The highest occupied molecular orbitals (HOMO) and lowest unoccupied molecular orbitals (LUMO) have importance for a molecule and are named frontier molecular orbitals (FMOs). FMO analysis is very important as it determines the material's electronic behavior and optical characteristics.^50,51^ It also provides information about the material's electrical conductivity, electron distribution, stability, and sensing response.^52^ The reactivity and interaction of the gas with the nanocluster and many other properties are obtained from the HOMO–LUMO energy gap (*E*_g_). The information about the transfer of charges and reactivity response of the system can be explored by the *E*_g_ values. The least energy gap values express a high electronic distribution from the donor to acceptor orbitals, which enhance the electrical conductivity of the corresponding complex. It has been also evidenced from the literature survey that the smaller *E*_g_ represents higher electrical conductivity, higher electron distribution, less stability, higher sensitivity, and *vice versa*.^53^

**Fig. 4:**
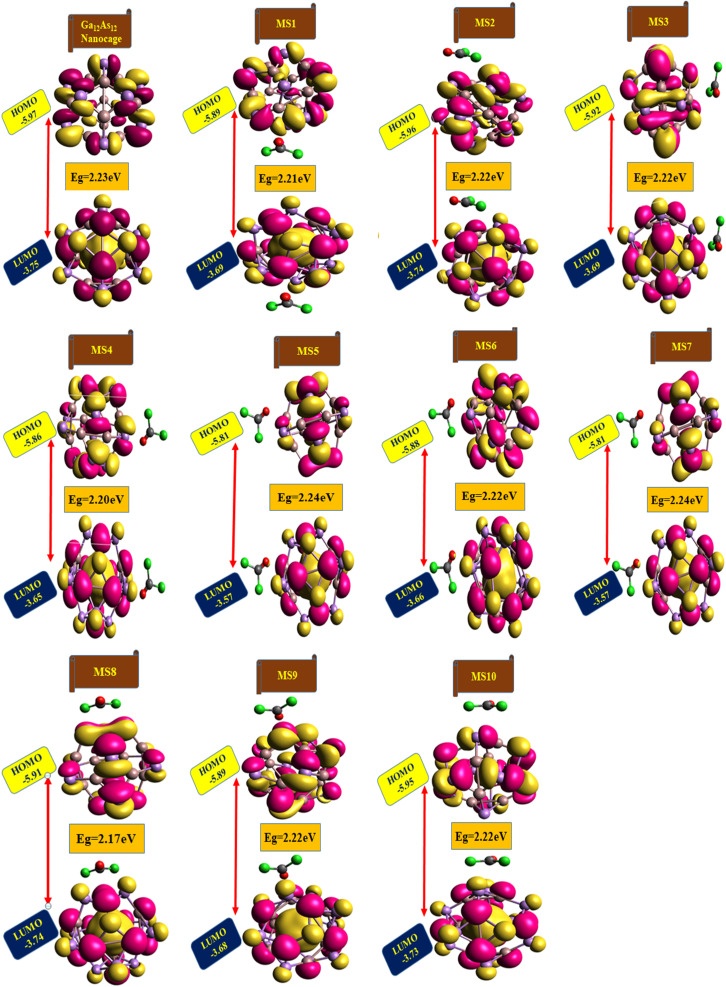
The HOMO and LUMO plots along with their calculated energy gap (*E*_g_) values for the studied nanocluster and complexes.

**Fig. 5:**
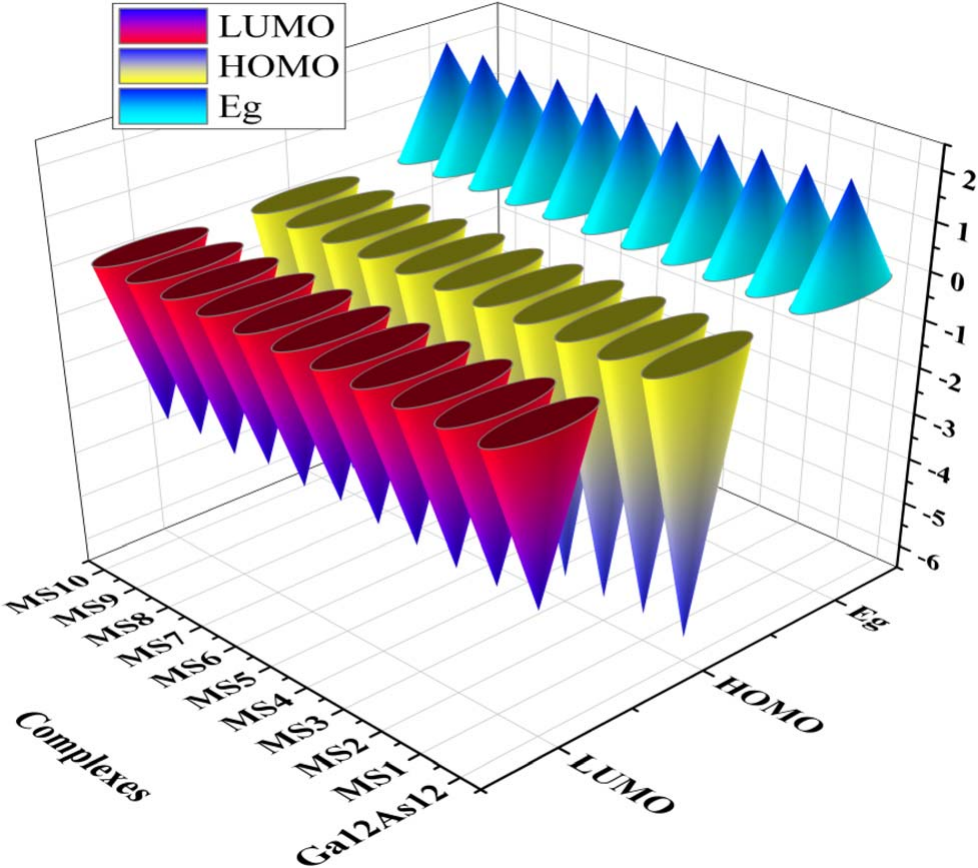
The graphical representation of HOMO/LUMO.

For the current investigation, the graph expressing the HOMO and LUMO relation of the complexes along with energy gap is shown in Fig. 5. It has been observed that the complex MS8 having less energy gap indicates high electronic distribution, less stability, high electrical conductivity, and high sensitivity, while complexes MS5 and MS7 have higher energy gap and exhibit less electronic distribution, high stability, low electrical conductivity, and low sensitivity as compared to all other complexes. The FMO orbitals of the nanocluster and complexes have been obtained by the Avogadro software, and these are shown in Fig. 4 with the corresponding energy values and *E*_g_ values for each complex. Similar to the *E*_g_ values, the Fermi level energy and work function values also provide information about the stability and reactivity of the system. The work function is the amount of required energy for the loss of an electron from the Fermi level, where the Fermi level energy (*E*_f_) is the amount of energy occupied by an electron at absolute zero temperature and can be calculated from the FMO orbitals, as given in eqn (4).^54^4
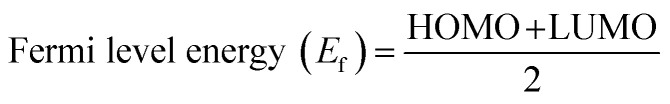


The *E*_f_ and work function (*Φ*) values for the studied complexes are also affected due to the interaction of gas and are found to be different from the isolated nanocluster values. The *E*_f_ is calculated as the average of HOMO and LUMO energy values, and the work function (*Φ*) is taken as the negative of the Fermi level energy value because the electrostatic potential energy is equal to zero.^55^ The relation between work function and Fermi level energy is given by eqn (5)

5 *Φ* = *V*_el_(+,∞) − *E*_f_

where *V*_el_(+,∞) is electrostatic potential energy and it is zero, *i.e.*, *V*_el_(+,∞) = 0, *Φ* = −*E*_f_

Literature review has shown that the variation in the work function (*Φ*) values due to the interaction of gas produces a sound by influencing the gate voltage to detect gas.^56^ Also, the changes in the work function values demonstrate the transfer of charges between phosgene gas and the studied nanocluster. The minimum work function and Fermi level energy values are experienced by the complexes MS5 and MS7, as shown in Table 3.

**Table 3:** The calculated energy values of the highest occupied molecular orbital (*E*_HOMO_) and lowest unoccupied molecular orbital (*E*_LUMO_), energy gap (*E*_g_), Fermi level energy (*E*_f_), and work function (Φ) of the nanocluster and complexes^a^

Name	*E*_HOMO_	*E*_LUMO_	*E*_g_	*E*_f_	*Φ*
Ga_12_As_12_	−5.97	−3.75	2.23	−4.86	4.86
MS1	−5.89	−3.69	2.21	−4.79	4.79
MS2	−5.96	−3.74	2.22	−4.85	4.85
MS3	−5.92	−3.69	2.22	−4.80	4.80
MS4	−5.86	−3.66	2.20	−4.76	4.76
MS5	−5.81	−3.57	2.24	−4.69	4.69
MS6	−5.88	−3.66	2.22	−4.77	4.77
MS7	−5.81	−3.57	2.24	−4.69	4.69
MS8	−5.91	−3.74	2.17	−4.82	4.82
MS9	−5.89	−3.68	2.22	−4.78	4.78
MS10	−5.95	−3.73	2.22	−4.84	4.84

a All factors are in electron volt (eV).

### Global indices of reactivity

The HOMO and LUMO energies provide information about various reactive, electrical, and optical properties of a molecule, such as electron affinity (*A*), ionization potential (*I*), and electronegativity (*χ*), which is equal to the negative of chemical potential (*μ*) expressed by Koopmans' theorem.^57,58^ The chemical hardness (*η*) is associated with the difference in electron affinity, and ionization potential and inverse of chemical hardness provide electrophilicity index (*ω*).^59^ The softness (*S*) and Δ*N*_max_ are also obtained by the equations provided in the ESI.† Δ*N*_max_ is the maximum amount of electronic charge accepted by the system.^60^ These properties are calculated and listed in Table 4. The softness and hardness of the compound are obtained from the energy gap (*E*_g_). The large energy gap represents the hardness of the compound, while the small energy gap shows the soft compound, and these energy gaps also provide information about the reactivity and sensitivity of the compound.^61^ The capability of the species to attract electrons toward itself is called electronegativity (*A*) while the ionization potential (*I*) is the required amount of energy for the loss of electrons from the surface of the compound.

**Table 4:** The calculated global reactivity parameters of the studied system^a^

Name	*E*_g_	*I*	*A*	*η*	*μ*	*s*	*ω*	Δ*N*_max_
Ga_12_As_12_	2.23	5.97	3.75	1.11	−4.86	0.45	10.61	4.37
MS1	2.21	5.89	3.69	1.10	−4.79	0.45	10.38	4.34
MS2	2.22	5.96	3.74	1.11	−4.85	0.45	10.62	4.38
MS3	2.22	5.92	3.69	1.11	−4.80	0.45	10.38	4.32
MS4	2.20	5.86	3.66	1.10	−4.76	0.45	10.30	4.33
MS5	2.24	5.81	3.57	1.12	−4.69	0.45	9.82	4.19
MS6	2.22	5.88	3.66	1.11	−4.77	0.45	10.23	4.29
MS7	2.24	5.81	3.57	1.12	−4.69	0.45	9.82	4.19
MS8	2.17	5.91	3.74	1.09	−4.82	0.46	10.71	4.44
MS9	2.22	5.89	3.68	1.11	−4.78	0.45	10.32	4.31
MS10	2.23	5.95	3.73	1.11	−4.84	0.45	10.54	4.35

a Here, *E*_g_ = energy gap, *I* = ionization potential, *A* = electron affinity, *η* = chemical hardness, *μ* = chemical potential, *s* = chemical softness, *ω* = electrophilicity index, *N* = electronic charge density. All parameters are in eV.

The literature survey shows that the system having higher values of ionization potential and chemical hardness indicates less reactivity and less sensitivity. In contrast, the system indicates high sensitivity and reactivity and less stability because of low ionization potential values and chemical hardness. Similar results have been observed in view of the chemical softness as the system indicates high sensitivity and less stability by having higher values of chemical softness and *vice versa*. Also, the electrophilicity index (*ω*) values for the nanocluster and the complexes demonstrate the capability of the fragment to attain electrons. It also provides information about the stabilization energy to gain many electrons by the chemical species.^62,63^ In the current study, complex MS8 indicates comparatively higher sensitivity and reactivity because of the low chemical hardness (1.09 eV), high chemical softness (0.46 eV), and high electrophilicity index (10.71 eV) among all the other complexes of the studied system to attain the high capability to attain electrons and high electronic distribution. In comparison, complexes MS5 and MS7 have high chemical hardness (1.12 eV), low chemical softness (0.45 eV), and low electrophilicity index (9.81 eV) and therefore indicate less sensitivity among all other complexes. This information is also correlated with FMO analysis results and expresses the better sensing response of the nanocluster. Because the energy gap (*E*_g_) of the HOMO and LUMO orbitals of the nanocluster has been calculated to be 2.23 eV, the interaction of phosgene gas on the surface of the nanocluster reduces the energy gap to 2.22, 2.21, 2.20, and 2.17 eV on different adsorption sites. The reduction in the energy gap values expresses the enhanced interactions and reactivity of the Ga_12_As_12_ nanocluster toward phosgene gas.

### NBO analysis

This analysis indicates natural bond orbitals (NBO) using stabilization energy named as 2^nd^ order perturbation energy.^64^ This energy is useful for studying intermolecular and intramolecular charge transfer.^65^ The information about the distribution of electrons between atoms within the molecular bonds was developed by Weinhold *et al.*^66^ The information about the type of orbitals, interactions nature, and occupancy level present in between the occupied and virtual Lewis s orbitals are provided by NBO analysis.^42,67^ The stabilization energy is mathematically represented by eqn (6).6
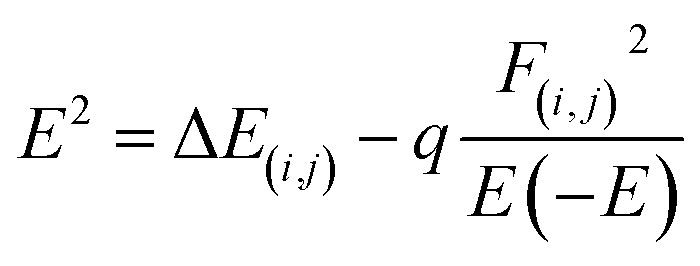


The diagonal elements are represented by *E*_*i*_ and *E*_*j*_. The Fock matrix and donor occupancy are denoted by *F*(*i*,*j*) and *q*, respectively. The important transitions providing stabilization energy to the investigated systems are presented in Table 5 while the remaining transitions are mentioned in Table S2 (ESI†). RY (Rydberg) and Cr (center core pair) interaction values are not mentioned in the NBO data of the studied system because these values represent loosely bonded and weak interactions. For complex MS1, the prominent and highest stabilization energy values are 38.88 kcal mol^−1^ and 21.69 kcal mol^−1^ corresponding to the LP(O_28_) → σ*(C_25_–Cl_27_) and LP(Cl_26_) → π*(C_25_–O_28_) interactions, while the highest stabilization energy values for complex MS2 are 42.15 kcal mol^−1^ and 21.22 kcal mol^−1^, associated with by LP(O_28_) → σ*(C_25_–Cl_26_) and LP(Cl_27_) → π*(C_25_–O_28_) interactions, respectively.

**Table 5:** The highest second order perturbation energy (*E*^2^) of the studied system calculated by DFT at the B3LYP-D3/6-31G(d,p) level of theory

Type	Donor	Type	Acceptor	*E* ^2^ (kcal mol^−1^)	*E*(*j*) − *E*(*i*)	*F*(*i*,*j*)
MS1						
LP	O_28_	σ*	C_25_–Cl_27_	38.88	0.39	0.112
LP	Cl_26_	π*	C_25_–O_28_	21.69	0.29	0.073
MS2						
LP	O_28_	σ*	C_25_–Cl_26_	42.15	0.37	0.115
LP	Cl_27_	π*	C_25_–O_28_	21.22	0.3	0.073
MS3						
LP	O_28_	σ*	C_25_–Cl_26_	8.4	0.39	0.112
LP	Cl_26_	π*	C_25_–O_28_	3.39	0.28	0.075
MS4						
LP	O_28_	σ*	C_25_–Cl_27_	38.74	0.39	0.113
LP	Cl_27_	π*	C_25_–O_28_	23.33	0.28	0.075
MS5						
LP	O_28_	σ*	C_25_–Cl_27_	36.76	0.42	0.113
LP	Cl_26_	π*	C_25_–O_28_	23.41	0.29	0.076
MS6						
LP	O_28_	σ*	C_25_–Cl_26_	38.56	0.4	0.114
LP	Cl_27_	π*	C_25_–O_28_	24.14	0.28	0.075
MS7						
LP	O_28_	σ*	C_25_–Cl_27_	36.53	0.42	0.113
LP	Cl_26_	π*	C_25_–O_28_	25.62	0.27	0.077
MS8						
LP	As_13_	σ*	Ga_6_–As_13_	6.05	0.73	0.06
LP	Cl_26_	π*	C_25_–O_28_	19.66	0.31	0.071
MS9						
LP	O_28_	σ*	C_25_–Cl_27_	37.95	0.39	0.111
LP	Cl_26_	π*	C_25_–O_28_	20.89	0.3	0.073
MS10						
LP	O_28_	σ*	C_25_–Cl_26_	41.87	0.37	0.114
LP	Cl_27_	π*	C_25_–O_28_	21.37	0.29	0.073

The highest stabilization energy values for complex MS3 are 8.4 kcal mol^−1^ corresponding to the LP(O_28_) → σ*(C_25_–Cl_26_) interactions while the highest *E*^2^ values for complex MS4 are 38.74 kcal mol^−1^ and 23.33 kcal mol^−1^ associated with by the (O_28_) → σ*(C_25_–Cl_27_) and LP(Cl_27_) → π*(C_25_–O_28_) interactions, respectively. Similarly, the highest stabilization energy values for complex MS5 associated with LP(O_28_) → σ*(C_25_–Cl_27_) and LP(Cl_26_) → π*(C_25_–O_28_) interactions are 36.76 kcal mol^−1^ and 23.41 kcal mol^−1^ and for complex MS6 are 38.56 kcal mol^−1^ and 24.14 kcal mol^−1^ as a result of LP(O_28_) → σ*(C_25_–Cl_26_) and LP(Cl_27_) → π*(C_25_–O_28_) interactions, respectively. For complex MS7, the prominent and highest *E*^2^ values are 36.53 kcal mol^−1^ and 25.62 kcal mol^−1^ corresponding to the LP(O_28_) → σ*(C_25_–Cl_27_) and LP(Cl_26_) → π*(C_25_–O_28_) interactions, respectively, while the highest stabilization energy values for complex MS8 are 19.66 kcal mol^−1^, associated with LP(As_13_) → σ*(Ga_6_–As_13_) interactions. The complex MS9 with LP(O_28_) → σ*(C_25_–Cl_27_), LP(Cl_26_) → π*(C_25_–O_28_) interactions has the highest *E*^2^ values, which are 37.95 kcal mol^−1^ and 20.89 kcal mol^−1^, while for complex MS10, they are 41.87 kcal mol^−1^ and 21.37 kcal mol^−1^ from the LP(O_28_) → σ*(C_25_–Cl_26_) and LP(Cl_27_) → π*(C_25_–O_28_) interactions, respectively.

The above-mentioned transitions (lone pair to surface) ensure the effective delocalization of the electrons of chlorine and oxygen atom of phosgene to the entire studied system. These results also explore that the donor–acceptor interactions are stabilized by intermolecular charge transfer (ICT) and different kinds of non-covalent interactions (NCI). These interactions and transfer of charges in between the molecules are because of the electron delocalization of the oxygen and the chlorine lone pair of the phosgene to the sigma and pi anti-bonding orbitals of the studied nanocluster. For these interactions, the complexes MS2 and MS10 have the highest stabilization energies, as mentioned in Table 5. The charge transfer transitions from LP to the ring provide the clue of binding, interaction, and sensing capability. Because of these bond evidences, it can be explored that the NCI and ICT network is present in the studied system, which represents a synchronism of NBO that results with NCI analysis and QTAIM analysis.

### QTAIM analysis

The analysis based on the quantum theory of atoms in a molecule, abbreviated as QTAIM, is useful for the topological study of the system. The QTAIM theory has been derived from Bader's theory.^68^ Its purpose was to investigate the molecule more deeply because the geometrical and electronic investigation of the system does not provide enough information to study the intermolecular interactions of the system. The information about the critical point properties of the system is provided by QTAIM analysis. The formation of complexes after interactions have been investigated by QTAIM analysis and the investigation data obtained are given in Table 6. The bond critical point, all electrons density *ρ*(*r*), electron density Laplacian, energy density, electronic charge density, and Hamiltonian and Lagrangian kinetic energies are the topological parameters denoted by BCP, *ρ*(*r*), *∇*^2^*ρ*(*r*), *E*(*r*), *V*(*r*), *H*(*r*), and *G*(*r*), respectively, and obtained by QTAIM analysis.^69,70^*∇*^2^*ρ*(*r*) and *ρ*(*r*) is used to determine the critical bond points that represent the intermolecular bonds by the sharing of electrons between the atoms in a molecule.^71^

**Table 6:** The topological parameters of the studied system after the interaction of phosgene gas. All the values are in atomic unit (a.u.)

Interaction	BCP	*ρ*(*r*)	*∇* ^2^ *ρ*(*r*)	*G*(*r*)	*K*(*r*)	*V*(*r*)	*H*(*r*)	|*V*(*r*)|/*G*(*r*)	ELF	*ε*
MS1										
Cl_27_–As_21_	40	0.00651	0.01905	0.00373	−0.00104	−0.00269	0.00104	0.72237	0.02952	0.55317
As_23_–O_28_	48	0.00920	0.02787	0.00615	−0.00082	−0.00533	0.00082	0.86667	0.03425	0.79641
As_21_–Cl_26_	62	0.00341	0.01999	0.00392	−0.00108	−0.00284	0.00108	0.72543	0.03119	0.35509
O_28_–As_19_	71	0.01001	0.02974	0.00660	−0.00083	−0.00577	0.00083	0.87406	0.03911	0.80730
MS2										
As_19_–Cl_27_	41	0.00619	0.01930	0.00369	−0.00113	−0.00256	0.00113	0.69334	0.02555	0.22728
Cl_27_–As_20_	61	0.00718	0.02189	0.00426	−0.00121	−0.00306	0.00121	0.71704	0.00121	0.07491
As_13_–C_25_	67	0.00812	0.02509	0.00497	−0.00130	−0.00366	0.00130	0.73743	0.03453	1.00327
Ga_2_–Cl_26_	80	0.00680	0.01686	0.00360	−0.00062	−0.00298	0.00062	0.82793	0.03633	3.53518
MS3										
As_22_–O_28_	45	0.00831	0.02506	0.00552	−0.00075	−0.00477	0.00075	0.86448	0.03043	0.04651
Cl_27_–Ga_10_	52	0.00768	0.01911	0.00411	−0.00067	−0.00344	0.00067	0.83784	0.04164	0.94279
O_28_–Als_23_	74	0.00822	0.02690	0.00537	−0.00135	−0.00402	0.00135	0.74792	0.03090	1.07179
MS4										
Ga_11_–Cl_26_	47	0.00713	0.01810	0.00384	−0.00069	−0.00315	0.00069	0.82149	0.03734	1.79382
As_22_–O_28_	73	0.01013	0.03043	0.00686	−0.00075	−0.00610	0.00075	0.89027	0.03784	0.41201
As_23_–O_28_	74	0.01021	0.03058	0.00689	−0.00076	−0.00613	0.00076	0.89022	0.03846	0.40887
MS5										
O_28_–Ga_3_	43	0.02119	0.06849	0.01752	0.00040	−0.01792	−0.00040	1.02292	0.06593	0.03493
As_16_–Cl_26_	68	0.00685	0.02071	0.00402	−0.00116	−0.00287	0.00116	0.71274	0.02995	0.19027
As_15_–Cl_26_	69	0.00714	0.02150	0.00419	−0.00118	−0.00302	0.00118	0.71880	0.03161	0.18004
MS6										
Ga_3_–O_28_	48	0.01447	0.03910	0.00986	0.00008	−0.00994	−0.00008	1.00846	0.05889	0.82473
As_14_–Cl_27_	68	0.00689	0.02115	0.00411	−0.00118	−0.00293	0.00118	0.71305	0.02929	0.19529
As_15_–Cl_27_	71	0.00750	0.02313	0.00454	−0.00124	−0.00330	0.00124	0.72593	0.03190	0.24332
Cl_27_–As_17_	87	0.00603	0.01886	0.00359	−0.00113	−0.00246	0.00113	0.68639	0.02480	0.32665
MS7										
O_28_–Ga_6_	54	0.02204	0.07190	0.01849	0.00051	−0.01900	−0.00051	1.02766	0.06743	0.01111
As_19_–Cl_26_	74	0.00716	0.02172	0.00424	−0.00119	−0.00304	0.00119	0.71862	0.03136	0.16373
As_18_–Cl_26_	78	0.00717	0.02173	0.00424	−0.00119	−0.00305	0.00119	0.71863	0.03136	0.16392
MS8										
As_18_–Cl_26_	37	0.00565	0.01598	0.00314	−0.00086	−0.00227	0.00086	0.72558	0.02607	2.79972
As_18_–O_28_	41	0.00692	0.02124	0.00453	−0.00078	−0.00375	0.00078	0.82827	0.02466	1.02710
O_28_–Ga_6_	52	0.00654	0.01944	0.00411	−0.00075	−0.00337	0.00075	0.81851	0.02480	0.09686
O_28_–As_19_	57	0.00693	0.02126	0.00454	−0.00078	−0.00376	0.00078	0.82846	0.02468	1.01979
Cl_27_–As_19_	69	0.00565	0.01598	0.00314	−0.00086	−0.00228	0.00086	0.72583	0.02609	2.82783
MS9										
O_28_–Ga_6_	49	0.01322	0.03723	0.00910	−0.00021	−0.00889	0.00021	0.97728	0.05143	0.38964
As_19_–Cl_27_	65	0.00687	0.02059	0.00405	−0.00109	−0.00296	0.00109	0.72987	0.02994	0.64918
Cl_27_–As_18_	69	0.00502	0.01475	0.00283	−0.00085	−0.00198	0.00085	0.69850	0.02165	0.62355
MS10										
Cl_26_–Ga_11_	43	0.00716	0.01739	0.00373	−0.00062	−0.00312	0.00062	0.83499	0.04002	1.65260
C_25_–As_22_	51	0.00807	0.02531	0.00502	−0.00131	−0.00370	0.00131	0.73860	0.03330	0.78816
Cl_27_–As_23_	71	0.00726	0.02172	0.00425	−0.00118	−0.00306	0.00118	0.72096	0.03265	0.47826

It has been shown in Table 6 that *∇*^2^*ρ*(*r*) for all the complexes is positive. The positive Laplacian electron density indicates the strong electrostatic bond between the two bonded atoms.^72^ The all-electron density *ρ*(*r*) is used to determine the strength of the chemical bond between the gas and the cage. Its positive values show the closed shell interactions, and the higher value of all electron density values indicates the greater strength of the chemical bond and *vice versa*.

The comparatively strong chemical bond has been observed in complex MS7 and the weak chemical bond in complex MS8 due to the comparatively higher and lower values of electron density, respectively, while all the other complexes have intermediate values of electron density, as shown in Table 6. The division of the chemical bond of the complexes into strong covalent, partial covalent, and non-covalent is achieved by the values of both *∇*^2^*ρ*(*r*) and *H*(*r*). For the strong covalent, *∇*^2^*ρ*(*r*) < 0, *H*(*r*) < 0. For the partial covalent, *∇*^2^*ρ*(*r*) > 0, *H*(*r*) < 0, and for non-covalent, *∇*^2^*ρ*(*r*) > 0, *H*(*r*) > 0.

It has been shown in Table 6 that the complexes MS5, MS6, and MS7 indicate partially covalent interactions while all the other complexes indicate weak covalent or non-covalent interactions. Furthermore, the energy densities of the bonds explain the nature and strength of interactions in the complex.^73^ The ratio between Hamiltonian kinetic energy values and absolute values of electronic charge density is represented as |*V*(*r*)|/*G*(*r*). The ratio |*V*(*r*)|/*G*(*r*) less than 1 indicates the presence of ionic bond or weak interactions (van der Waals interactions), while its value greater than 1 and less than 2 show mixed type of interactions. If the value of the ratio is greater than 2, then it indicates covalent bond.^74^

**Fig. 6:**
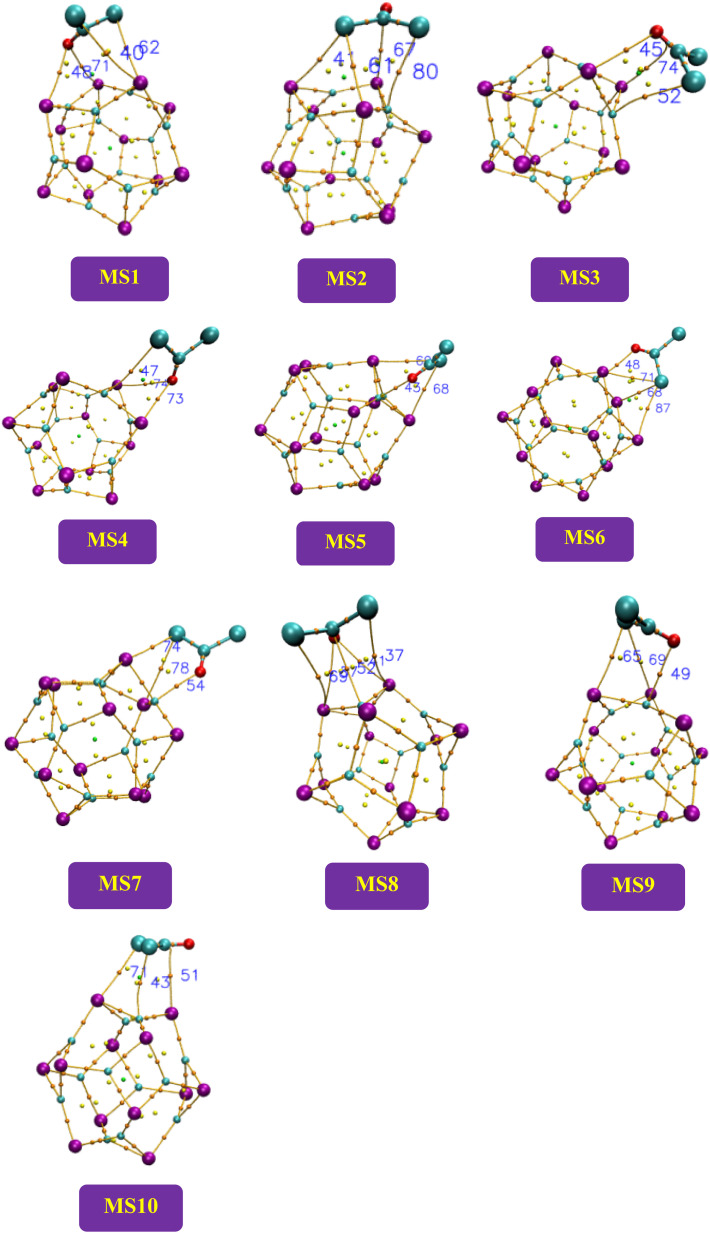
The schematic structures of the studied complexes by QTAIM analysis to represent bond critical points between the nanocluster and the adsorbate.

In the current study, complexes MS1 to MS4 and complexes MS8 to MS10 have less than 1 values of the ratio |*V*(*r*)|/*G*(*r*), and these complexes indicate van der Waals interactions, while the complexes MS5, MS6, and MS7 have mixed and covalent nature of interactions as the value of the ratio is >1 for these complexes (Fig. 6).

Another parameter, ellipticity, defines the stability of the interactions as its value > 1 represents the instability of the structure while its value < 1 shows the strength of the structure and interactions. In the current work, the ellipticity values of the complexes of the studied system ranges from 0.0111 to 3.5351 a.u. The maximum number of critical points are represented by complex MS8, indicating more interaction between the surface and the adsorbate. Another tool electron localization function (ELF) is useful for covalent bond analysis. The values of ELF range from 0.5 to 1.00, indicating the localization of bonding and non-bonding electrons, while its values < 0.5 indicate delocalized electrons.^75^ It is observed from Table 6 the values of ELF for all the complexes of the studied system are less than 0.5, and the electrons are delocalized for the studied system. All the above parameters indicate the excellent performance of the surface as an adsorbent material for sensing phosgene gas.

### Density of states

**Fig. 7:**
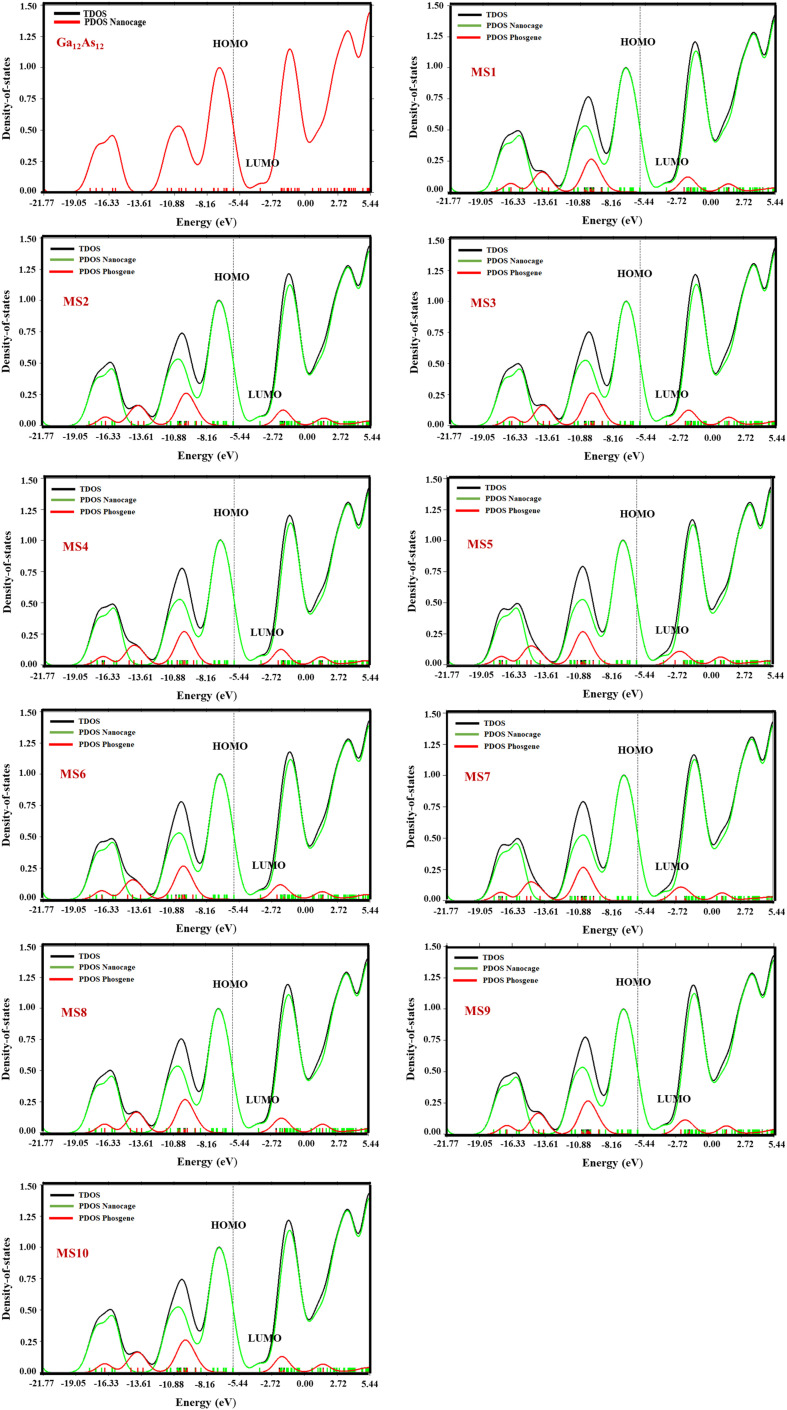
The calculated TDOS and PDOS plots of the studied complexes by DFT at the B3LYP-D3/6-31G(d,p) level of theory.

The important parameter of solid physics, through which numbers of states are described in the unit intervals of energy for the provided chemical system, is called density of states. The DOS graph is useful for analyzing the nature of electronic structure with the configuration of molecular orbitals with their proposed energies. The Multiwfn software was employed to calculate the DOS of the Ga_12_As_12_ complexes. The comparison between the DOS plot of the nanocluster and the DOS plots of its complexes with phosgene gas demonstrates the sensitivity of complexes toward phosgene gas and the variation in the complexes' electronic properties. By the interaction of the nanocluster and the gas for each complex, a few new energy states appeared around *E*_f_, which caused an increase and decrease in the energy gap (*E*_g_) values, as shown in Fig. 7.

The decrease in the *E*_g_ values for complexes MS1, MS4, and MS8 has been calculated as 0.02 eV, 0.03 eV, and 0.05 eV, respectively, while for complexes MS2, MS3, MS6, MS9, and MS10, it is 0.01 eV, but the increase in the *E*_g_ values for complexes MS5 and MS7 has been calculated as 0.01 eV. It has been shown that the maximum variation in the *E*_g_ value is observed for complex MS8, which represents the maximum conductivity and sensitivity of the studied system.

### NCI analysis

Non-covalent bonds are analyzed by applying the Multiwfn software. Hydrogen bonding, van der Waals interactions, and electrostatic interactions are those mechanisms through which non-covalent interactions are carried out. In order to predict weak interactions, NCI analysis of basic functions such as electron density, reduced density gradient, and Hessian 2^nd^ density eigenvalue is utilized. The information about the type of interactions is given by isosurface plots that involve two functions, namely, the eigenvalue of Hessian 2^nd^ density and electron density plotted against RDG.

By the isosurface plot, the nature of interactions is defined based on the eigenvalue. The negative values of *λ*_2_(*r*)*ρ*(*r*) and the high value of electron density represent strong non-covalent interactions, such as hydrogen bonding through the blue region, while the positive value of *λ*_2_(*r*)*ρ*(*r*) through the red region represents weak non-covalent interactions such as steric effect and reduced electron density.

**Fig. 8:**
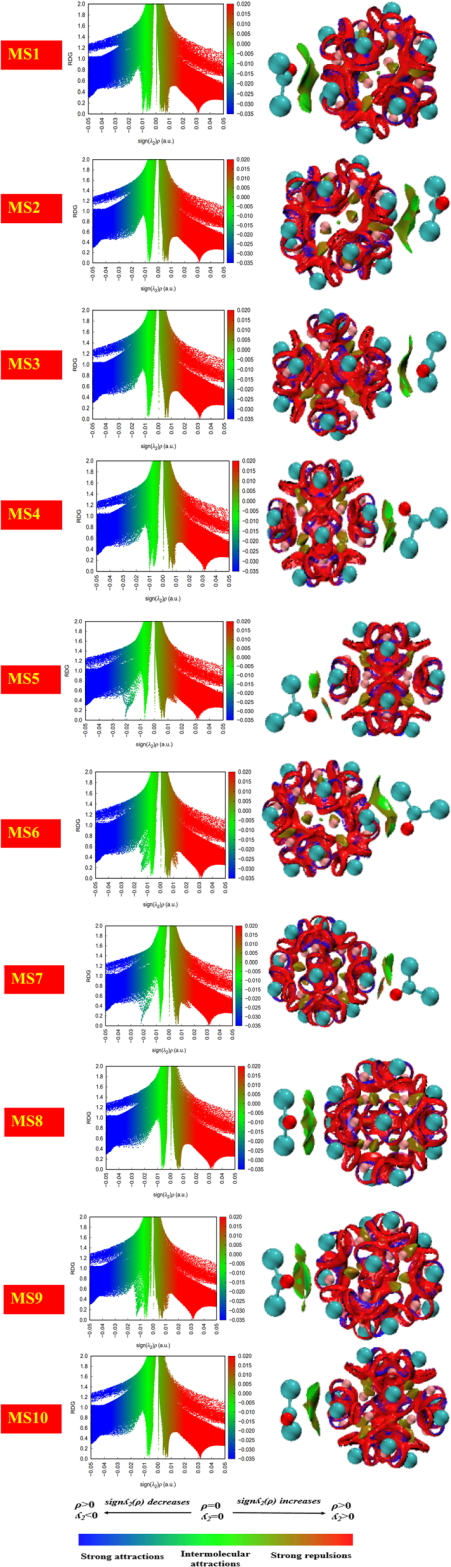
The pictorial display of the non-covalent interactions of the studied system.

In the case of the green region, the value of *λ*_2_(*r*)*ρ*(*r*) is zero, and it represents comparatively weak intermolecular interactions such as van der Waals interactions.^47,76^ In the current work, it has been evident through the plots shown in Fig. 8 that the existence of the blue region in between the nanocluster represents strong non-covalent interactions.

The existence of the green region in between phosgene gas and the nanocluster for all complexes indicates the presence of van der Waals interactions. Further, repulsive interactions such as the steric effect were also observed in between the atoms of the nanocluster and indicated by the red region.

### Molecular electrostatic potential analysis

**Fig. 9:**
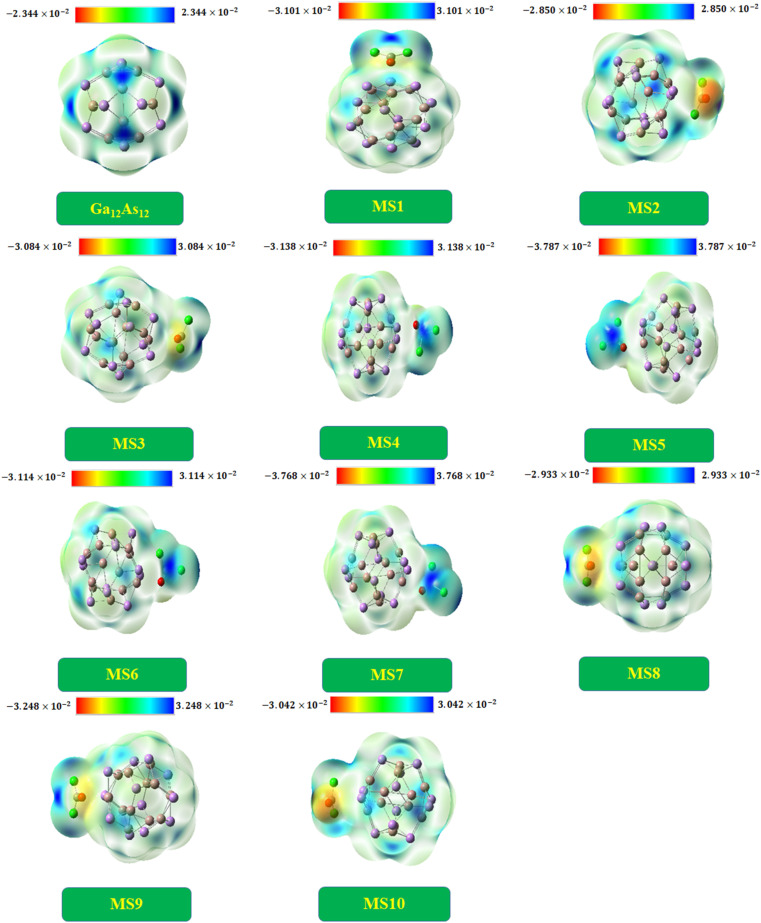
The MEP diagrams of the Ga_12_As_12_ nanocluster and complexes under study.

In order to analyze the limit of charge distribution in a molecule, molecular electrostatic potential (MEP) analysis is very useful. The system's physiochemical properties, such as chemical reactivity, partial charges, and dipole moment, are correlated with the system's geometry by MEP analysis.^29^ The analysis was done by DFT at the B3LYP-D3/6-31G(d,p) level of theory. The charge distribution is disclosed in Fig. 9.

Commonly, the electropositive end (electron deficient area) is represented by the blue region, while the electronegative end (electron-rich area) is characterized by the yellow region and the green area existing between two extreme regions indicates the mean potential in the web version.^77^ The isolated nanocluster indicates equal charge distribution with both charges at a similar extent because of the symmetrical geometry. The nanocluster fixed with phosgene gas produces insignificant charge separation by decreasing the blue region intensity on the nanocluster and shifting toward the gas in the case of complexes MS4, MS5, MS6, and MS7. In the case of other complexes, the intensity of the yellow region increases on the gas, as shown in Fig. 9.

The pure Ga_12_As_12_ nanocluster has zero dipole moment because of the same number of electronegative and electropositive atoms, but after the interaction of phosgene gas, the variation in dipole moment takes place due to the shifting of (blue and yellow region) charges between the nanocluster and the gas. In MEP analysis, to understand the interaction strength of phosgene gas with the nanocluster, the charge distribution is also correlated with dipole moment (*D*_m_), which is further supported by *Q*_T_ (the calculated net charge on phosgene gas) analysis. For all complexes of the studied system, the irregular behavior of *D*_m_ was observed. The maximum value of *D*_m_ was observed for complex MS7, and it causes the shifting of the blue region toward the phosgene gas, as shown in the MEP plots. The 2^nd^ largest *D*_m_ value has been noted for complex MS5, and some changes have been observed in its MEP plot.

The MEP plots of the complexes MS2 and MS10 indicate similar potential, and a similar change in charge distribution has been observed because of the low and moderate values of *D*_m_ and *E*_INT_, respectively. In the same context, different charge distribution has been observed due to different *D*_m_ and *E*_INT_ values and different interaction distances. This result also correlates with NBO findings.

The calculated dipole moment values and *Q*_T_ values are found to be consistent with each other, as shown in Table 7. The highest *Q*_T_ value has been observed for complex MS7 with large *D*_m_ and *E*_INT_ values. The variation in *Q*_T_ and *D*_m_ values for different complexes has been observed because of extra charge distribution and due to which the shifting of blue and yellow regions has been observed. These observations indicate the excellent response of the nanocluster in the sensing and detection of phosgene gas.

**Table 7:** The calculated excitation energy (Δ*E*), maximum absorbance (*λ*_max_) along with oscillator strength (*f*), and dipole moment (*D*_m_) of the studied system and the net charge on phosgene gas (*Q*_T_) after adsorption

System	Δ*E* (eV)	*D*_m_ (debye)	*Q*_T_	*λ*_max_ (nm)	*f*
Ga_12_As_12_	2.25	0.000		551.95	0.0007
MS1	2.25	1.698	0.058	549.92	0.0007
MS2	2.23	0.799	0.062	556.63	0.0008
MS3	2.26	1.510	0.054	548.68	0.0008
MS4	2.26	2.337	0.058	547.68	0.0008
MS5	2.28	3.638	0.093	542.74	0.0010
MS6	2.25	2.091	0.077	551.83	0.0008
MS7	2.29	3.722	0.096	542.17	0.0010
MS8	2.21	1.290	0.049	562.11	0.0009
MS9	2.23	1.818	0.060	556.56	0.0009
MS10	2.25	0.916	0.060	551.56	0.0008

### UV-Vis analysis

The UV-Vis spectra of all the complexes of the Ga_12_As_12_ nanocluster with phosgene gas have been calculated utilizing the TD-DFT method at the B3LYP-D3/6-31G(d,p) level of theory. The adsorption behavior of the isolated nanocluster has been calculated and compared with its complexes after the adsorption of phosgene gas to demonstrate its sensing capability based on the UV-Vis adsorption technique. The strong overlapping due to the interacting moieties is indicated by enhanced absorption wavelength along with oscillator strengths (*f*), as mentioned in Table 7.

**Fig. 10:**
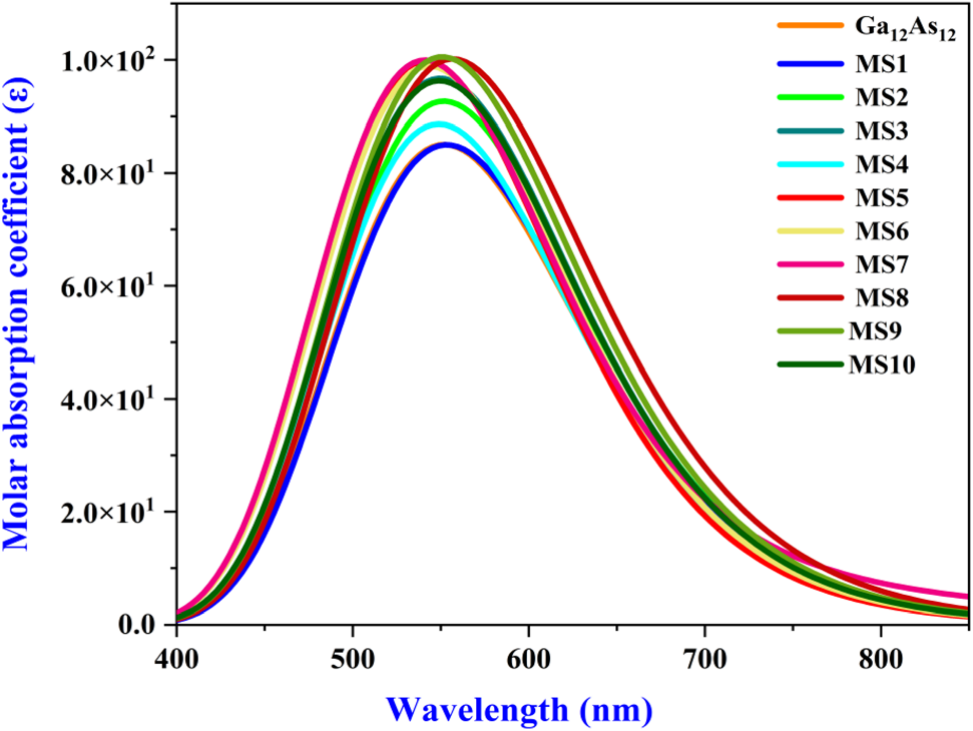
The UV-Vis adsorption spectrum of the studied system.

In the current work, the change in the adsorption spectra of the Ga_12_As_12_ nanocluster due to the presence of phosgene gas has been explained. The combined adsorption spectrum of the nanocluster with its complexes has been shown in Fig. 10. The combined adsorption spectrum of the studied system is composed of eleven peaks located in the wavelength range of 400–850 nm.

The maximum absorbance of the isolated nanocluster is at 551.95 nm. The maximum absorbance of complexes MS2, MS9, and MS8 is red-shifted because of the shifting to longer wavelengths 556.63 nm, 556.56 nm, and 562.11 nm, respectively, because of nanocluster interaction with phosgene gas. The red-shifted absorbance indicates the reduced band gap and enhanced electrical conductivity and the sensing response of the studied system.^78^ Similar complexes have minimum excitation energy (Δ*E*) and high conductivity values. Herein, the comparatively maximum absorbance wavelength and minimum excitation energy value is represented by complex MS8 while the minimum absorbance wavelength and maximum excitation energy values are represented by complexes MS5 and MS7. This result is correlated with FMO analysis and chemical reactivity indices values where MS8 show the least energy gap, high softness, high electrophilicity index, and maximum conductivity and express high sensing response. It can also be noted the *λ*_max_ is also blue-shifted for all the other complexes because of shifting toward shorter wavelength (551.83–542.17 nm), and for complex MS1, the interaction of phosgene gas with the nanocluster creates considerable variation in the adsorption spectrum. The variation in molar adsorption coefficient values and red/blue shift absorbance wavelength after phosgene adsorption indicate that the Ga_12_As_12_ nanocluster has the potential as an excellent sensor for phosgene gas.

### Sensing mechanism

The main purpose of the work is to investigate the capability of the nanocluster to detect and sense phosgene gas. The sensing mechanism is a parameter that characterizes conductivity and resistance changes before and after gas interaction. The sensing response of the nanocluster is associated with the change in the conductivity of the nanocluster and the conductivity of its complexes. It could be determined by the given eqn (7)7
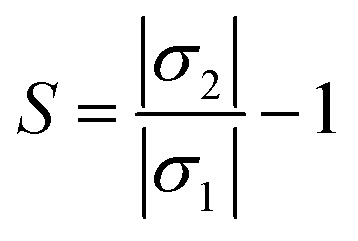
where *σ*_2_ is the electrical conductivities of the complexes and *σ*_1_ is the electrical conductivity of the nanocluster. The electrical conductivity is inverse of the electrical resistance, and both these parameters and recovery time is used to determine the sensing response.

### Electrical conductivity

The movement of electrons from the valence bond to the conduction bond is termed as electrical conductivity.^79^ The changes in the electrical conductivity of the complexes are due to their different electronic characteristics. The electrical conductivity is determined by eqn (8).^55^8
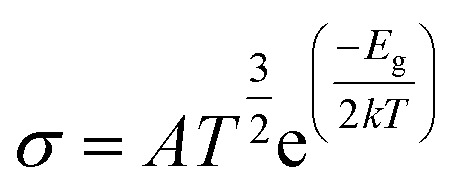
where *A*, *T*, and *k* are Richardson constant (6 × 10^5^), working temperature (298 K), and Boltzmann constant (8.318 × 10^−3^ kJ mol^−1^ K^−1^), respectively.^80^ The given equation relates the electrical conductivity with the HOMO–LUMO energy gap values, and this shows that the increase in the energy gap values causes a decrease in the electrical conductivity of the complexes. After the adsorption of the gas, the energy gap values are changed due to variations in the HOMO and LUMO values of the complexes.

**Fig. 11:**
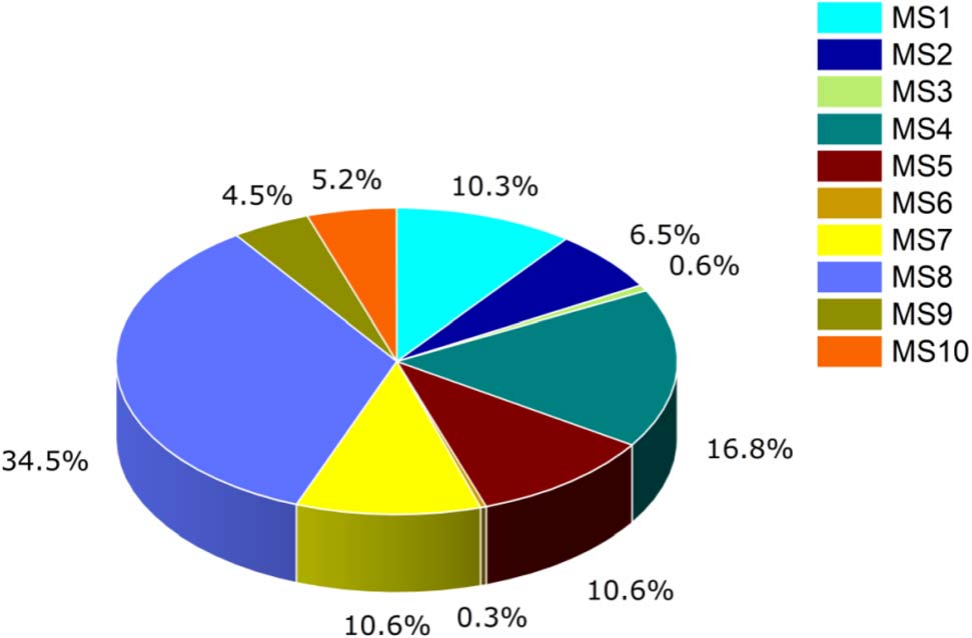
The sensing response of the studied system.

The electrical conductivity of the studied system has been calculated to determine the sensing response of the complexes. The sensitivity of all the complexes of the nanocluster with phosgene gas has been calculated and shown in Table 8. Complex MS8, having less energy gap and high electrical conductivity, respond excellently in sensing, and this result is also correlated with FMO analysis, global indices of reactivity, and UV-Vis analysis. It can be evidence to express the mechanistic sensing capability of the nanocluster. The sensing response of the studied system is displayed by Fig. 11.

**Table 8:** The electrical conductivity, recovery time, and sensing response of the system under study calculated by DFT at the B3LYP-D3/6-31G(d,p) level of theory^a^

Name	*E*_INT_	*E*_g_	*σ*	*τ*	*S*
MS1	−3.8154	2.21	1.98 × 10^9^	4.66 × 10^−12^	0.0032
MS2	−4.4772	2.22	1.97 × 10^9^	6.09 × 10^−12^	0.0020
MS3	−3.1981	2.22	1.97 × 10^9^	3.63 × 10^−12^	0.0002
MS4	−3.1309	2.20	1.98 × 10^9^	3.54 × 10^−12^	0.0052
MS5	−4.5565	2.24	1.96 × 10^9^	6.28 × 10^−12^	−0.0033
MS6	−4.6578	2.22	1.97 × 10^9^	6.55 × 10^−12^	0.0001
MS7	−4.5920	2.24	1.96 × 10^9^	6.37 × 10^−12^	−0.0033
MS8	−2.5608	2.17	1.99 × 10^9^	2.42 × 10^−12^	0.0107
MS9	−2.1920	2.22	1.97 × 10^9^	2.81 × 10^−12^	0.0014
MS10	−3.2691	2.22	1.97 × 10^9^	3.74 × 10^−12^	0.0016

a Here, *E*_g_ = energy gap, *E*_INT_ = counterpoise corrected interaction energy in kcal mol^−1^, *τ* = recovery time in second, *σ* = complexes electrical conductivity, *S* = sensitivity.

### Recovery time

The time a sensor utilizes to return to its original shape after the adsorption of the material is named its recovery time and denoted by *τ*. It indicates the sensing performance of the material. The surface having a shorter recovery time performs better to sense the material and *vice versa*. It can be calculated by the given eqn (9).^81^9
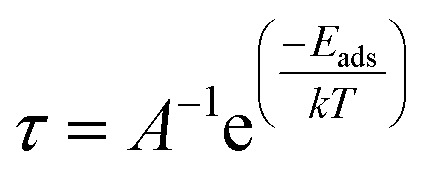
where *A* is the vibrational frequency of the complex, which is 10^12^ s^−^^1^, and *T* and *k* are working temperature and Boltzmann constant, respectively.^82^*E*_ads_ is the adsorption energy of the complex, and its negative values represent an exothermic reaction. The more negative values of *E*_ads_ indicate strong interactions between the gas and the nanocluster.

The adsorption strength also affects the sensing capability of the material because it makes for difficult desorption of the gas and long recovery time. In the present work, the recovery time of the complexes has been calculated by putting counterpoise corrected interaction energy (*E*_INT_) values in eqn (2) to investigate the absolute relation of recovery time and sensitivity, as shown in Table 8. Among all the complexes, complex MS8 has the shortest recovery time and represents the highest reactivity and sensitivity. This investigation is also correlated with UV-Vis analysis, chemical reactivity of indices, FMO analysis, and conductivity response to describe the mechanistic sensing response of the nanocluster.

### Thermodynamics analysis

The interaction of work and heat with chemical reactions and variations in the physical state through the law of thermodynamics is studied by thermodynamic analysis (TDA). This mathematical method is applied to study the spontaneous and non-spontaneous behavior of the system and chemical equations.^42,43^ The current work is concerned with enthalpy change and Gibbs' free energy change. The relation between the internal energy of the system and enthalpy change is represented by the equation Δ*H* = *Q* + *PV*. The positive and negative values of the enthalpy change depend upon the absorbance and release of heat. The enthalpy change is positive due to the absorbance of heat by the system, and the reaction is endothermic. In contrast, the enthalpy change is negative due to the release of heat by the system, and the response is exothermic. The relation of enthalpy change with Gibbs' free energy change is expressed by eqn (10)

10 Δ*G* = Δ*H* − *T*Δ*S*

where *T* is the system's temperature and Δ*S* is the entropy change of the system. The information related to the spontaneity of the system is provided by the change in Gibbs' free energy. The spontaneous reaction and non-spontaneous reaction are represented by the negative and the positive values of Δ*G*, respectively. When the value of Gibbs' free energy change is zero, then the reaction is at an equilibrium state, has the tendency to maximum entropy, and is expressed by eqn (11).

11 Δ*H* = *T*Δ*S*

The enthalpy changes and Gibbs' free energy change of the complexes have been calculated from the optimized geometries of the complexes using the following eqn (12)–(15).

12 Δ*H*^0^(298 K) = ∑productΔ_f_*H*^0^prod.(298 K) − ∑reactantΔ_f_*H*^0^react.(298 K)

13 Δ_f_*H*^0^(298 K) = ∑(*E*_0_ + *H*_Corr_)product − ∑(*E*_0_ + *H*_Corr_)reactants

14 Δ*G*^0^(298 K) = ∑productΔ_r_*G*^0^prod.(298 K) − ∑reactantΔ_r_*G*^0^react.(298 K)

15 Δ_r_*G*^0^(298 K) = ∑(*E*_0_ + *G*_Corr_)product − ∑(*E*_0_ + *G*_Corr_)reactants

where *E*_0_, *H*_Corr_, *G*_Corr_, Δ_f_*H*^0^, and Δ_r_*G*^0^ represent electronic energy, thermal correction for *H*, thermal correction for *G*, standard enthalpy changes, and change in the Gibbs' free energy of formation, respectively. The other thermodynamic properties of the system, such as zero-point energy, heat capacity, and entropy change, have been calculated by DFT at the B3LYP-D3/6-31G(d,p) level of theory at a constant working temperature (298 K).

In the current work, the calculated enthalpy changes and Gibbs' free energy changes of the nanocluster and phosgene gas complexes are clearly shown in Table S3 (ESI†) in a detailed form. The enthalpy of formation for all the studied complexes MS1, MS2, MS3, MS4, MS5, MS6, MS7, MS8, MS9, and MS10 is −22.8441, −21.1185, −19.0917, −17.5242, −19.3157, −20.0674, −19.4174, −20.2733, −20.6397, and −19.3653 kcal mol^−1^, respectively. The standard enthalpy is negative as the heat is released by the system due to the interaction between the nanocluster and gas, indicating that the reaction is exothermic. Also, the values of Δ_r_*G*^0^ calculated for the complexes of the studied system after phosgene adsorption are −12.8537 kcal mol^−1^ for MS1, −11.3383 kcal mol^−1^ for MS2, −10.7183 kcal mol^−1^ for MS3, and −8.1776 kcal mol^−1^ for MS4, −10.7008 kcal mol^−1^ for MS5, −10.3895 kcal mol^−1^ for MS6, −10.5922 kcal mol^−1^ for MS7, −10.2741 kcal mol^−1^ for MS8, −11.1293 kcal mol^−1^ for MS9, and −10.4127 kcal mol^−1^ for MS10. All the values of Δ_r_*G*^0^ are also negative and show that the reaction is spontaneous.

## Conclusion

The capability of the semiconductor Ga_12_As_12_ nanocluster to sense and adsorb the warfare agent phosgene gas is examined employing comprehensive DFT insights. Utilizing computational tools, geometry optimization, adsorption studies, frontier molecular orbitals analysis, global indices of reactivity, NBO analysis, topological QTAIM analysis, NCI analysis, MEP analysis, UV-Vis analysis, thermodynamics analysis, and the density of states of ten selected complexes of the studied system has been investigated. The adsorption studies represent the maximum adsorption response of the nanocluster with −21.34 ± 2.7 kcal mol^−1^ adsorption energy and the least interaction distance (3.05 ± 0.5 Å) with phosgene gas. FMO analysis revealed that complex MS8 has the least energy gap (2.17 eV) and indicates less stability and high conductivity. DOS analysis has also been performed in support of FMO analysis, which explores the distribution patterns of HOMO–LUMO orbitals. The global indices of reactivity indicate comparatively the highest softness (0.46 eV), least hardness (1.09 eV), high electrophilicity index (10.71 eV), and high reactivity of complex MS8 among all the complexes of the studied system. The ionization potential values of the studied system have been reduced by the adsorption of phosgene gas, and these factors indicate the enhanced reactivity and sensitivity of the studied system. Furthermore, NBO results explore that the donor–acceptor interactions are stabilized by intermolecular charge transfer (ICT) and different kinds of non-covalent interactions. These interactions and transfer of charges in between the molecules are because of electron delocalization of the oxygen and chlorine lone pair of the phosgene to the sigma and pi anti-bonding orbitals of the studied nanocluster because of high stabilization energy values. The topological parameters of QTAIM analysis provide information about the presence of partially covalent and non-covalent interactions and delocalized electrons between the nanocluster and phosgene gas. NCI analysis indicates green region existence, and its results are also compatible with the information obtained by NBO and QTAIM analysis. MEP analysis provides confirmation about the charge distribution by shifting the yellow region toward the nanocluster and shows that the calculated dipole moment and *Q*_T_ values of the studied system are found to be consistent with each other. UV-Vis analysis expresses the maximum absorbance (redshift) and minimum excitation energy of complex MS8, and this information is also correlated with the results of FMO analysis and global indices of reactivity. The sensing mechanism indicates the shortest recovery time (2.42 × 10^−12^), high conductivity (1.99 × 10^9^), and excellent sensing response (0.0107) of nanocluster's complex MS8 toward the phosgene gas. It is also correlated with FMO analysis, global indices of reactivity analysis, and UV-Vis analysis. Furthermore, thermodynamics analysis shows spontaneous thermodynamic behavior and exothermic interaction process of the studied nanocluster with phosgene gas. Thus, it can be concluded from all these investigations that the Ga_12_As_12_ nanocluster is a promising influential sensor for phosgene gas detection, MS8 has proven to be the best isomer of the studied system, and this research will emphasize the informative knowledge for experimental researchers to use Ga_12_As_12_ as a sensor for chemical defence and environmental monitoring in industry and military safety. It can be used further for chemical agent neutralization upon detection and forensic investigation to analyze traces of chemical warfare agent after chemical attack.

## Conflicts of interest

There are no conflicts to declare.

## Supplementary Material

RA-013-D3RA05086F-s001
