# Supplementary material for: Deciphering the electrochemical sensing capability of novel Ga12As12 nanocluster towards chemical warfare phosgene gas: insights from DFT
Source: RSC Adv. 2023 Oct 2;13(41):28885–903. doi: 10.1039/d3ra05086f (PMC10543987; doi:10.1039/d3ra05086f)
Supplement: RA-013-D3RA05086F-s001 [file RA-013-D3RA05086F-s001.pdf]

Electronic Supplementary Material (ESI) for RSC Advances.
This journal is © The Royal Society of Chemistry 2023

Deciphering the electrochemical sensing capability of novel $Ga_{12}As_{12}$ nanocluster towards chemical warfare phosgene gas: Insight from DFT

Muhammad Javed,[1] Muhammad Usman Khan,*[1] Riaz Hussain,*[1] Sarfraz Ahmed,[2] Tansir Ahamad,[3]

[1]*Department of Chemistry, University of Okara, Okara-56300, Pakistan*

[2]*Wellman Center for Photomedicine, Harvard Medical School, Massachusetts General Hospital, Boston, MA 02114, United States*

[3]*Department of Chemistry, College of Science, King Saud University, Saudi Arabia*

*** Corresponding authors E-mail addresses:**

- **Dr. Muhammad Usman Khan**

  E-mail ids: usman.chemistry@gmail.com; usmankhan@uo.edu.pk

- **Dr. Riaz Hussain**

  E-mail ids: riazhussain@uo.edu.pk

Table S1: The interaction distance of phosgene atoms with the most closed Gallium and Arsenide atoms of the nanocluster calculated at B3LYP-D3/6-31G(d, p) level of theory.

System	D(Ga)		D(As)	
**Surface- $Ga_{12}As_{12}$**	**IA**	**ID**	**IA**	**ID**
**MS1**	Ga_9-O_{28}	3.21	As_{19}-O_{28}	3.23
**MS2**	Ga_2-Cl_{26}	3.56	As_{13}-C_{25}	3.38
**MS3**	Ga_{10}-Cl_{27}	3.46	As_{22}-O_{28}	3.29
**MS4**	Ga_{11}-Cl_{26}	3.44	As_{22}-O_{28}	3.21
**MS5**	Ga_3-O_{28}	2.57	As_{14}-O_{28}	3.57
**MS6**	Ga_3-O_{28}	2.83	As_{15}-O_{28}	3.42
**MS7**	Ga_6-O_{28}	2.55	As_{13}-O_{28}	3.58
**MS8**	Ga_6-C_{25}	3.33	As_{19}-O_{28}	3.43
**MS9**	Ga_6-O_{28}	2.85	As_{13}-O_{28}	3.48
**MS10**	Ga_{11}-Cl_{26}	3.52	As_{22}-C_{25}	3.37

D (Ga) = distance of gas with closest Ga atom of the nanocluster, D (As) = distance of gas with closest As atom of the nanocluster, IA=interacted atoms, ID=interaction distance. The distance is measured in A^0.

Table S2: The calculated second order perturbation energy (**E^2 Kcal/mol)** for the remaining transitions of the studied system.

Type	Donor	Type	Acceptor	E^2 (kcal/mol)	E (j) – E (i)	F (i,j)
			**MS1**			
σ	Ga_4-As_{21}	σ *	Ga_5-As_{19}	9.31	0.51	0.062
σ	Ga_4-As_{21}	π*	C_{25}-O_{28}	0.27	0.35	0.009
π	C_{25}-O_{28}	π*	C_{25}-O_{28}	0.54	0.4	0.014
π	C_{25}-O_{28}	σ*	Ga_9-As_{18}	0.82	0.82	0.025
			**MS2**			
σ	Ga_4-As_{21}	σ*	Ga_5-As_{19}	9.67	0.5	0.062
σ	Ga_1-As_{13}	π*	C_{25}-O_{28}	0.11	0.37	0.006
π	C_{25}-O_{28}	π*	C_{25}-O_{28}	0.57	0.41	0.014
π	C_{25}-O_{28}	σ*	Ga_1-As_{13}	0.33	0.53	0.012
			**MS3**			
σ	Ga_{11}-As_{23}	σ*	Ga_{10}-As_{21}	9.81	0.5	0.063
σ	Ga_{10}-As_{23}	π*	C_{25}-O_{28}	0.4	0.36	0.011
π	C_{25}-O_{28}	π*	C_{25}-O_{28}	0.66	0.39	0.015

π	C_{25}-O_{28}	σ*	Ga_9-As_{23}	0.43	0.55	0.014
			**MS4**			
σ	Ga_{10}-As_{22}	σ*	Ga_{11}-As_{24}	10.11	0.5	0.064
σ	Ga_{10}-As_{23}	π*	C_{25}-O_{28}	0.09	0.34	0.005
π	C_{25}-O_{28}	π*	C_{25}-O_{28}	0.62	0.39	0.015
π	C_{25}-O_{28}	σ*	Ga_9-As_{23}	0.23	0.56	0.011
			**MS5**			
σ	Ga_3-As_{16}	σ*	Ga_{12}-As_{22}	9.99	0.51	0.064
σ	Ga_3-As_{15}	π*	C_{25}-O_{28}	0.09	0.35	0.005
π	C_{25}-O_{28}	π*	C_{25}-O_{28}	0.6	0.42	0.015
π	C_{25}-O_{28}	σ*	Ga_3-As_{15}	0.24	0.57	0.011
			**MS6**			
σ	Ga_3-As_{14}	σ*	Ga_3-As_{15}	9.85	0.53	0.065
σ	Ga_3-As_{15}	π*	C_{25}-O_{28}	0.07	0.34	0.004
π	C_{25}-O_{28}	π*	C_{25}-O_{28}	0.66	0.39	0.015
π	C_{25}-O_{28}	σ*	Ga_{12}-As_{15}	0.11	0.54	0.007
			**MS7**			
σ	Ga_6-As_{18}	σ*	Ga_9-As_{23}	9.76	0.51	0.064
σ	Ga_6-As_{18}	π*	C_{25}-O_{28}	0.11	0.33	0.005
π	C_{25}-O_{28}	π*	C_{25}-O_{28}	0.74	0.38	0.016
π	C_{25}-O_{28}	σ*	Ga_6-As_{18}	0.13	0.55	0.008
			**MS8**			
σ	Ga_9-As_{19}	σ*	Ga_6-As_{13}	11.09	0.49	0.066
σ	Ga_6-As_{13}	π*	C_{25}-O_{28}	0.1	0.43	0.006
π	C_{25}-O_{28}	σ*	Ga_5-As_{19}	0.21	0.58	0.01
			**MS9**			
σ	Ga_9-As_{18}	σ*	Ga_6-As_{13}	9.88	0.49	0.063
σ	Ga_6-As_{19}	π*	C_{25}-O_{28}	0.75	0.37	0.015
π	C_{25}-O_{28}	σ*	Ga_6-As_{18}	0.33	0.57	0.013
			**MS10**			
σ	Ga_{10}-As_{23}	σ*	Ga_{11}-As_{24}	9.87	0.5	0.063
σ	Ga_{11}-As_{22}	π*	C_{25}-O_{28}	0.2	0.36	0.008
π	C_{25}-O_{28}	π*	C_{25}-O_{28}	0.58	0.4	0.014
π	C_{25}-O_{28}	σ*	Ga_{12}-As_{22}	0.29	0.55	0.012

Table S3: Thermodynamics parameters of the studied system include heat capacity (C_V), entropy (S), electronic energy (E_0), zero-point energy (E_{ZPE}), Thermal correction for enthalpy (H_{corr}) and Gibbs energy (G_{corr}). All parameters are in Hartree. Here, $\Delta_f H^0$ and $\Delta_r G^0$ are enthalpy and Gibbs energy of formation at 298K temperature.

Parameters	$Ga_{12}As_{12}$	Phosgene	MS1
C_V	127.924	11.85	145.54
S	273.207	67.80	307.499
E_0	-49881.8826	-1033.7155	-50915.6365
E_{ZPE}	0.0257	0.0104	0.0372
E_{tot}	0.0697	0.0144	0.0870
H_{corr}	0.0707	0.0153	0.0880
G_{corr}	-0.0591	-0.0168	-0.0581
E_0+E_{ZPE}	-49881.8569	-1033.7050	-50915.5992
E_0+E_{tot}	-49881.8129	-1033.7010	-50915.5494
E_0+H_{corr}	-49881.8119	-1033.7001	-50915.5485
E_0+G_{corr}	-49881.9417	-1033.7323	-50915.6946
$\Delta_f H^0$ (298K)			-22.8441 kcal/mol
$\Delta_r G^0$ (298K)			-12.8537 kcal/mol

Parameters	$Ga_{12}As_{12}$	Phosgene	MS2
C_V	127.924	11.85	145.657
S	273.207	67.80	308.205
E_0	-49881.8826	-1033.7155	-50915.6337
E_{ZPE}	0.0257	0.0104	0.0371
E_{tot}	0.0697	0.0144	0.0870
H_{corr}	0.0707	0.0153	0.0879
G_{corr}	-0.0591	-0.0168	-0.0584
E_0+E_{ZPE}	-49881.8569	-1033.7050	-50915.5965
E_0+E_{tot}	-49881.8129	-1033.7010	-50915.5467
E_0+H_{corr}	-49881.8119	-1033.7001	-50915.5457
E_0+G_{corr}	-49881.9417	-1033.7323	-50915.6922
$\Delta_f H^0$ (298K)			-21.1185 kcal/mol
$\Delta_r G^0$ (298K)			-11.3382 kcal/mol

Parameters	$Ga_{12}As_{12}$	Phosgene	MS3
C_V	127.924	11.85	145.596
S	273.207	67.80	312.922
E_0	-49881.8826	-1033.7155	-50915.6305
E_{ZPE}	0.0257	0.0104	0.0370
E_{tot}	0.0697	0.0144	0.0870
H_{corr}	0.0707	0.0153	0.0879
G_{corr}	-0.0591	-0.0168	-0.0606
E_0+E_{ZPE}	-49881.8569	-1033.7050	-50915.5934
E_0+E_{tot}	-49881.8129	-1033.7010	-50915.5434
E_0+H_{corr}	-49881.8119	-1033.7001	-50915.5425
E_0+G_{corr}	-49881.9417	-1033.7323	-50915.6912
$\Delta_f H^0$ (298K)			-19.0916 kcal/mol
$\Delta_r G^0$ (298K)			-10.7183 kcal/mol

Parameters	$Ga_{12}As_{12}$	Phosgene	MS4
C_V	127.924	11.85	145.573
S	273.207	67.80	309.659
E_0	-49881.8826	-1033.7155	-50915.6279
E_{ZPE}	0.0257	0.0104	0.0370
E_{tot}	0.0697	0.0144	0.0869
H_{corr}	0.0707	0.0153	0.0878
G_{corr}	-0.0591	-0.0168	-0.0592
E_0+E_{ZPE}	-49881.8569	-1033.7050	-50915.5908
E_0+E_{tot}	-49881.8129	-1033.7010	-50915.5409
E_0+H_{corr}	-49881.8119	-1033.7001	-50915.5400
E_0+G_{corr}	-49881.9417	-1033.7323	-50915.6871
$\Delta_f H^0$ (298K)			-17.5241 kcal/mol
$\Delta_r G^0$ (298K)			-8.1775 kcal/mol

Parameters	$Ga_{12}As_{12}$	Phosgene	MS5
C_V	127.924	11.85	145.414
S	273.207	67.80	312.114
E_0	-49881.8826	-1033.7155	-50915.6309
E_{ZPE}	0.0257	0.0104	0.0371

**E_{tot}**	0.0697	0.0144	0.0870
**H_{corr}**	0.0707	0.0153	0.0880
**G_{corr}**	-0.0591	-0.0168	-0.0602
**E_0+E_{ZPE}**	-49881.8569	-1033.7050	-50915.5937
**E_0+E_{tot}**	-49881.8129	-1033.7010	-50915.5438
**E_0+H_{corr}**	-49881.8119	-1033.7001	-50915.5428
**E_0+G_{corr}**	-49881.9417	-1033.7323	-50915.6911
**$\Delta_f H^0$ (298K)**			-19.3157 kcal/mol
**$\Delta_r G^0$ (298K)**			-10.7007 kcal/mol

**Parameters**	**$Ga_{12}As_{12}$**	**Phosgene**	**MS6**
**C_V**	127.924	11.85	145.449
**S**	273.207	67.80	308.549
**E_0**	-49881.8826	-1033.7155	-50915.6321
**E_{ZPE}**	0.0257	0.0104	0.0373
**E_{tot}**	0.0697	0.0144	0.0870
**H_{corr}**	0.0707	0.0153	0.0880
**G_{corr}**	-0.0591	-0.0168	-0.0585
**E_0+E_{ZPE}**	-49881.8569	-1033.7050	-50915.5948
**E_0+E_{tot}**	-49881.8129	-1033.7010	-50915.5450
**E_0+H_{corr}**	-49881.8119	-1033.7001	-50915.5440
**E_0+G_{corr}**	-49881.9417	-1033.7323	-50915.6906
**$\Delta_f H^0$ (298K)**			-20.0674 kcal/mol
**$\Delta_r G^0$ (298K)**			-10.3895 kcal/mol

**Parameters**	**$Ga_{12}As_{12}$**	**Phosgene**	**MS7**
**C_V**	127.924	11.85	145.406
**S**	273.207	67.80	311.407
**E_0**	-49881.8826	-1033.7155	-50915.6310
**E_{ZPE}**	0.0257	0.0104	0.0371
**E_{tot}**	0.0697	0.0144	0.0870
**H_{corr}**	0.0707	0.0153	0.0880
**G_{corr}**	-0.0591	-0.0168	-0.0599
**E_0+E_{ZPE}**	-49881.8569	-1033.7050	-50915.5939
**E_0+E_{tot}**	-49881.8129	-1033.7010	-50915.5440

E_0+H_{corr}	-49881.8119	-1033.7001	-50915.5430
E_0+G_{corr}	-49881.9417	-1033.7323	-50915.6910
Δ_fH^0 (298K)			-19.4173 kcal/mol
Δ_rG^0 (298K)			-10.5922 kcal/mol

Parameters	$Ga_{12}As_{12}$	Phosgene	MS8
C_V	127.924	11.85	145.542
S	273.207	67.80	307.471
E_0	-49881.8826	-1033.7155	-50915.6324
E_{ZPE}	0.0257	0.0104	0.0372
E_{tot}	0.0697	0.0144	0.0870
H_{corr}	0.0707	0.0153	0.0880
G_{corr}	-0.0591	-0.0168	-0.0580
E_0+E_{ZPE}	-49881.8569	-1033.7050	-50915.5951
E_0+E_{tot}	-49881.8129	-1033.7010	-50915.5453
E_0+H_{corr}	-49881.8119	-1033.7001	-50915.5444
E_0+G_{corr}	-49881.9417	-1033.7323	-50915.6905
Δ_fH^0 (298K)			-20.2732 kcal/mol
Δ_rG^0 (298K)			-10.2740 kcal/mol

Parameters	$Ga_{12}As_{12}$	Phosgene	MS9
C_V	127.924	11.85	145.505
S	273.207	67.80	309.109
E_0	-49881.8826	-1033.7155	-50915.6329
E_{ZPE}	0.0257	0.0104	0.0371
E_{tot}	0.0697	0.0144	0.087039
H_{corr}	0.0707	0.0153	0.0879
G_{corr}	-0.0591	-0.0168	-0.0588
E_0+E_{ZPE}	-49881.8569	-1033.7050	-50915.5958
E_0+E_{tot}	-49881.8129	-1033.7010	-50915.5459
E_0+H_{corr}	-49881.8119	-1033.7001	-50915.5450
E_0+G_{corr}	-49881.9417	-1033.7323	-50915.6918
Δ_fH^0 (298K)			-20.6397 kcal/mol
Δ_rG^0 (298K)			-11.1293 kcal/mol

Parameters	$Ga_{12}As_{12}$	Phosgene	MS10
C_V	127.924	11.85	145.659
S	273.207	67.80	310.981
E_0	-49881.8826	-1033.7155	-50915.630953
E_{ZPE}	0.0257	0.0104	0.037098
E_{tot}	0.0697	0.0144	0.087031
H_{corr}	0.0707	0.0153	0.087975
G_{corr}	-0.0591	-0.0168	-0.059782
E_0+E_{ZPE}	-49881.8569	-1033.7050	-50915.593855
E_0+E_{tot}	-49881.8129	-1033.7010	-50915.543922
E_0+H_{corr}	-49881.8119	-1033.7001	-50915.542978
E_0+G_{corr}	-49881.9417	-1033.7323	-50915.690735
$\Delta_f H^0$ (298K)			-19.3652 kcal/mol
$\Delta_r G^0$ (298K)			-10.4127 kcal/mol

Equations (1-5) used to calculate the global indices of reactivity for the studied system.

$$\eta = \frac{I - A}{2} \quad (1)$$

$$\mu = \frac{-(I + A)}{2} \quad (2)$$

$$\omega = \frac{\mu 2}{2\eta} \quad (3)$$

$$s = \frac{1}{2\eta} \quad (4)$$

$$\Delta N_{max} = \frac{-\mu}{\eta} \quad (5)$$
